# Low-dimensional assemblies of metal-organic framework particles and mutually coordinated anisotropy

**DOI:** 10.1038/s41467-022-31651-3

**Published:** 2022-07-09

**Authors:** Dengping Lyu, Wei Xu, Jae Elise L. Payong, Tianran Zhang, Yufeng Wang

**Affiliations:** Department of Chemistry, The University of Hong Kong, Pokfulam Road, 999077 Hong Kong SAR, China

**Keywords:** Metal-organic frameworks, Self-assembly, Colloids

## Abstract

Assembling metal-organic framework (MOF)-based particles is an emerging approach for creating colloidal superstructures and hierarchical functional materials. However, realization of this goal requires strategies that not only regulate particle interactions but also harness the anisotropic morphologies and functions of various frameworks. Here, by exploiting depletion interaction induced by ionic amphiphiles, we show the assembly of a broad range of low-dimensional MOF colloidal superstructures, including 1D straight chains, alternating or bundled chains, 2D films of hexagonal, square, centered rectangular, and snowflake-like architectures, and quasi-3D supercrystals. With well-defined polyhedral shapes, the MOF particles are mutually oriented upon assembly, producing super-frameworks with hierarchically coordinated crystallinity and micropores. We demonstrate this advantage by creating functional MOF films with optical anisotropy, in our cases, birefringence and anisotropic fluorescence. Given the variety of MOFs available, our technique should allow access to advanced materials for sensing, optics, and photonics.

## Abstract (web summary)

Colloidal self-assembly is a powerful strategy for designing materials, and MOFs offer wide structural and functional diversity. Here, authors present the self-assembly of MOF microcrystals using depletion interactions to form low-dimensional MOF colloidal superstructures with anisotropic properties.

## Introduction

Colloidal self-assembly is a powerful strategy for designing new materials, whereby nano- or micro-scale particles self-organize into superstructures with emerging properties^1,2^. Diverse assemblies and applications have been realized by using particles composed of polymers, noble metals, and metal oxides^3–6^. A crucial step to expand the toolkit for colloidal assembly is to exploit other functional compositions. A notable example is metal-organic framework (MOF), a versatile type of hybrid materials featuring three-dimensional (3D) crystalline network of metal nodes and organic struts^7,8^. With periodically ordered micropores capable of hosting guest molecules, MOFs are highly functional for molecular storage, separation, and catalysis^7–9^.

Synthesizing MOF as uniform particles and assembling them into colloidal superstructures open promising avenues for material design and fabrication. On the one hand, MOF particles are polyhedral crystallites, whose anisotropic shapes favor directional bonding, a sought-after quality in modern colloid science for producing low-coordinated and open structures^10–12^. The shape and size of MOF particles are also widely tunable, for example, allowing access to polyhedral particles in the micrometer scale (which are less investigated and are considered different from commonly studied nanoparticles in assembly)^13,14^. On the other hand, the molecular crystallinity and anisotropic property of the framework, originally restricted within individual crystallite, may be mutually synchronized via assembly and particle alignment^14^. This can lead to super-frameworks, which, in contrast to conventional MOF bulk materials containing crystallites of random sizes and shuffled orientations, may unlock MOF’s advanced properties for sensing, microelectronics, and optics^15^.

Despite the enormous potentials, harnessing the rich and unique information encoded in diverse MOF systems (with regards to their diverse types, shapes, sizes, and functions) and expressing them into new colloidal structures and functional properties remain limited. So far, several approaches have been explored to assemble MOF particles, using capillary force^16–18^, DNA hybridization^19^, polymer matrix^20^, electric field^21^, and more recently liquid bridging^22^. Although assemblies have been achieved, these methods either require extensive surface functionalization, focus on densely packed nanometer-sized particles, or produce imperfectly aligned structures. Among various technical issues, MOF particles generally suffer from low colloidal (and chemical) stability^23,24^, which prevents their assembly into equilibrium structures. Consequently, the MOFs employed and the assembled structures have been restricted to a few kinds, and more importantly, the progress for integrating MOF functions with their superstructures towards advanced materials have been impeded.

Here, we present the self-assembly of MOF microcrystals using depletion interaction induced by ionic amphiphiles. This strategy is surprisingly easy to operate, applicable to common MOFs, and promote equilibrium assembly that acknowledges the particles’ polyhedral geometries and micrometer sizes. A wide range of colloidal superstructures are therefore achieved, including not only quasi-3D/3D lattices, but also unexpected low-dimensional, low-coordinated assemblies such as one-dimensional (1D) chains and two-dimensional (2D) films of various types. These structures are highly tunable and in situ observations rationalize their formation kinetics. The superstructures feature mutually oriented particles, and hence coordinated frameworks and aligned molecular micropores, serving as supreme platforms for creating hierarchical anisotropic materials. We show, for example, that colloidal films of selected MOFs display birefringent properties. The film can also host guest dye molecules with coordinated orientation to enable anisotropic fluorescence. With this approach, we envisage the realization of other advanced materials and properties.

## Results

### General strategy for self-assembly

Depletion interaction, an entropic effect induced by deletants in the form of non-adsorbing polymers, micelles, or nanoparticles, encourages short range attractive force between larger colloids. For polyhedral colloids, the interaction encourages particles contact in a face-to-face manner, which maximizes the system entropy and motivates shape-directed assembly. Although depletion force has been employed in other systems (e.g., lock and key colloids, silica cubes, patchy particles, and polyhedral nanocrystals)^25–28^, its use with MOFs has not been demonstrated. Such progress may have been delayed by challenges in dealing the special properties of MOF particles, for example, the low surface charges and exposed coordination sites (metal, ligand, or pores)^24^, which cause undesirable binding (between particles or between particle and depletant) and preclude equilibrium assembly.

We discover that simply adding small-molecule ionic amphiphiles such as cetyltrimethylammonium chloride (CTAC) and sodium dodecyl sulfate (SDS) works well for assembling MOF particles in an aqueous environment. Similar amphiphiles have been previously used in mediating the MOF assembly, yet they serve a distinct role^18^. In our case, CTAC (for example) can adsorb on MOF surface creating a protective coating, which ensures the colloidal stability while keeping their morphology intact (see characterization in Supplementary Fig. 1, in Supplementary Information). Simultaneously, it forms micellar nanoparticles acting as the depletants to exert depletion force on particles^27,29^. As we discuss below, this approach applies to common MOFs and produces superstructures with ease and fidelity. It allows us to thoroughly explore and harness the versatile features of MOF systems—their diverse types, anisotropic shapes, tunable sizes, and versatile functions—towards new superstructures and properties.

**Fig. 1:**
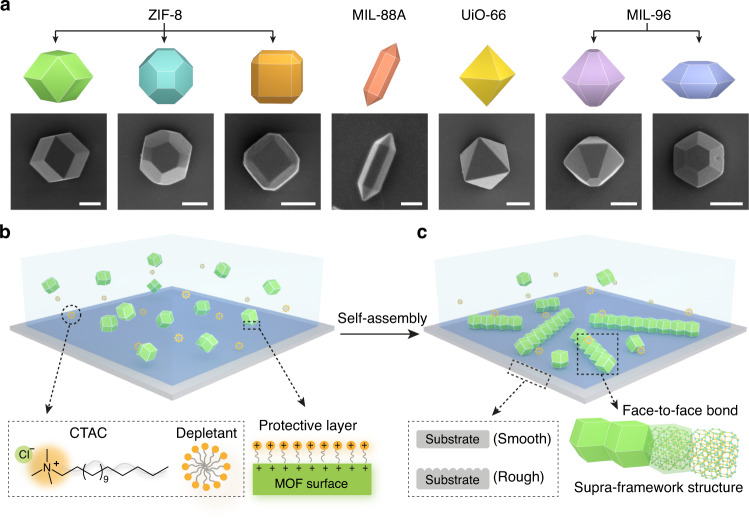
Strategy for assembling MOF microcrystals. **a** Cartoons (top panel) and scanning electron micrographs (SEM, bottom panel) showing microcrystals of common MOF families. Scale bars: 500 nm. **b**, **c** Schematics showing the self-assembly of MOF microcrystals via depletion interaction induced by ionic amphiphiles (e.g., cetyltrimethylammonium chloride, CTAC). Particles of a representative shape (rhombic dodecahedral, RD, green) are well dispersed in aqueous solution (light blue, **b**) and then assembled into low-dimensional MOF colloidal superstructures (or supra-framework structures) by establishing shape-directed face-to-face bonds (**c**). The molecular structure of CTAC, cartoon of the CTAC micelle as depletant, and illustration of protective surface layer are shown. The substrate of assembly (gray) with a smooth or rough surface influences the particle orientation and assembly outcome.

To implement, we synthesize monodisperse microcrystals (0.5–5.1 µm in size) based on ZIFs (Zeolitic Imidazolate Framework)^30^, MILs (Materials Institute Lavoisier)^31,32^ and UiOs (Universitetet I Oslo)^33^ (see Methods). They adopt a wide spectrum of polyhedral geometries and symmetries, as exhibited by cartoons and scanning electron micrographs (SEM) in Fig. 1a (see also Supplementary Fig. 2). Besides introducing interaction between particles, we also regulate (add or remove) the depletion attraction between particles and substrate, by using either a pristine smooth or a modified rough substrate (see Methods and Supplementary Fig. 3). When micrometer-sized MOF particles settle and assemble on a substrate, their orientation and available binding sites are controlled, which influences the directionality and dimension of the assembly. Our strategy is sketched in Fig. 1b, c and Supplementary Fig. 4a.

### Superstructure of ZIF-8 particles and dimension control in self-assembly

**Fig. 2:**
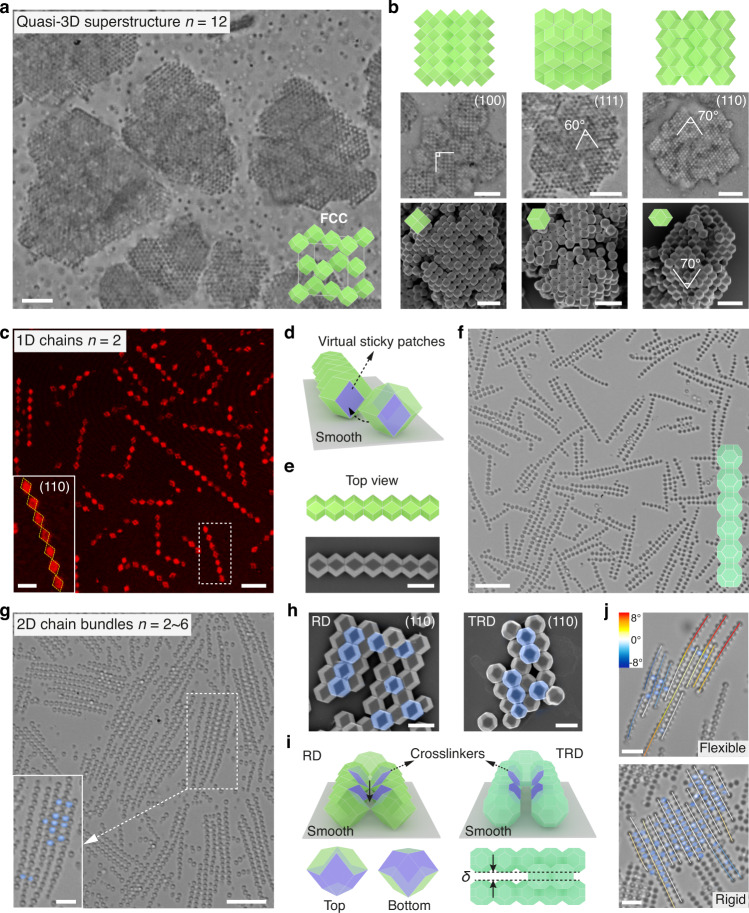
Self-assembly of ZIF-8 particles and dimension control. **a**, **b** Quasi-3D superstructures assembled from 0.9-µm ZIF-8 RD particles on a rough substrate. For each particle, the coordination number *n* = 12. Large-view optical microscope image of the assembled crystals with a face-centered cubic structure (FCC) is shown in (**a**). Cartoons, zoomed-in optical images and SEMs in (**b**) show (100)-, (111)-, and (110)-oriented colloidal crystals; the axial angles are labeled. **c**–**f** 1D chains (*n* = 2) by assembling ZIF-8 RD (**c**–**e**) or truncated rhombic dodecahedra (TRD) (**f**) particles on a smooth substrate. Reflected-light confocal microscopy image in (**c**) reveals the well-arrayed rhombic faces (inset, orange dashed lines) within the RD chain. Cartoon in (**d**) illustrates that the particles stand on the substrate by their (110) faces and contact with one another by virtual patches (purple) to form a chain. Cartoon and SEM image in (**e**) show the top view of a colloidal chain. **f** Cartoon and bright-field optical image of chains assembled by TRD particles. **g**–**j** 2D chain bundles (*n* = 2~6). Optical (**g**) and SEM images (**h**) show the structure of ZIF-8 chain bundles and highlight the crosslinker particles (in blue). Cartoons in (**i**) illustrate the binding and the corresponding particle facets (purple) between the chains (at bottom layer) and the crosslinkers (at upper layer). A gap with a width of *δ* is observed between the bridged chains of TRD particles. Optical images in (**j**) show the flexible and rigid chain bundles according to the number density of crosslinkers. Color of chain segment denotes the deviation in angles from straight chain (white line). Scale bars: 5 µm for microscope images in (**a**, **b**), 2 μm for SEMs in (**b**), 5 and 2 µm (inset) for (**c**), 1 µm for (**e**, **h**), 10 µm for (**f**), 10 and 5 µm (inset) for (**g**), and 3 µm for (**j**).

We demonstrate our strategy first with a common zeolitic framework, ZIF-8, whose microcrystals adopt various shapes such as (truncated) rhombic dodecahedra (TRD/RD) (Fig. 1a)^34,35^. Taking 0.9-µm RD particles as an example, they become well dispersed on mixing with CTAC (Supplementary Fig. 1g). The zeta potential increases from *ζ* = +15.8 mV to *ζ* = +56.0 mV. When an appropriate concentration of CTAC (4.0 mM) is used, the particles show reversible binding/dissociation signifying equilibrium assembly. Upon incubation (minutes to hours), big chunks of colloidal crystals consisting of tens of hundreds of particles are formed, as shown by optical microscope image in Fig. 2a and Supplementary Fig. 4. Owing to the micrometer scale particle size, the crystals are quasi-3D, each spanning tens of micrometers in width and several micrometers in height. The crystal structure is face-centered cubic (coordination number *n* = 12), whereby particles closely pack via a full face-to-face overlap. We note that, in forming these superlattices, a rough substrate is used to avoid its interaction with the particles. The (100)-, (111)-, and (110)-oriented supercrystals are observed in Fig. 2b.

In stark contrast, when 2.6-µm RD particles are assembled on a smooth substrate, straight 1D chains form (Fig. 2c). The particles first settle on the substrate and are confined in plane by sticking its rhombic (110) facet to the substrate with depletion interaction. As such, each particle only has two faces, labeled in purple in Fig. 2d, that remain geometrically eligible in plane for the preferred face-to-face binding, thus forming chains (*n* = 2). These two faces can be considered as virtual “sticky patches”, which are developed only in the relevant context. We use reflected-light confocal microscopy to image the particle faces in contact with the substrate (see Methods and Supplementary Fig. 5). As shown in Fig. 2c, the corresponding rhombic faces are clearly visible. Importantly, they are also mutually aligned, suggesting a strictly coordinated particle orientation. SEM imaging confirms the chain structure and the mentioned binding mode (Fig. 2e).

Similarly, the assembly of TRD particles also forms chains, when the additional square (100) facets are kept relatively small. The chains formed by 1.2-µm TRD particles (for example) are shown in Fig. 2f. Yet, they may further bind by their side (100) facets to form a 2D square lattice (Supplementary Fig. 6).

When the concentration of MOF particles is increased, we observe bundles consisting of multiple chains that are relatively aligned (Fig. 2g–j), formed when “crosslinker” particles bridge the 1D chains from the upper layer. The crosslinkers are particles escaping from or settling to the substrate by thermal motion (Supplementary Fig. 7). They can be distinguished either by optical contrast or by confocal fluorescence microscopy (Fig. 2g, inset and Supplementary Fig. 8). In such cases, the beveled side faces of the particles bind the crosslinker by forming four face-to-face bonds shown in Fig. 2i.

With a small number of crosslinker particles that scatter at random locations, the chain bundles can be regarded as porous 2D superstructure (*n* = 2~6). The structure formation is possible for both RD and TRD particles (SEM in Fig. 2h), while the TRD chains feature a larger separation distance *δ* due to truncation (denoted in Fig. 2i). With fewer crosslinkers, the chains become slightly flexible, whereas more crosslinkers produce rigid bundles. The flexibility of the chain segments is color-coded (Fig. 2j). The detail effect of particle concentration on the assembly is included in Supplementary Fig. 9.

Besides dimension control, another merit of our systems is the ability to easily observe the assembly kinetics in real time and space under the microscope. For example, the nucleation and growth of the quasi-3D structure are shown in Supplementary Movie 1. The assembly of 1.2-µm ZIF-8 TRD particles into chain is shown in Supplementary Movie 2. We have also studied ZIF-8 particles with cubic shapes. They assemble into 2D films of square superlattice and its variants; more details are included in Supplementary Fig. 10, Note 1, and Movies 3-4.

### Alternating chains and snowflake-like networks by MIL-88A particles

We extend our strategy to other MOFs. While the scheme proves generally applicable, we focus on MOFs that produce low-dimensional (1D and 2D) structures. 1D materials (chains and fibers) with anisotropic morphology can offer distinct mechanical, optical, and electronic properties, whereas 2D films are essential for their integration into functional devices^36–38^. Producing low-dimensional structures via assembly often requires highly directional colloidal interactions or relies on the use of external fields^11,39,40^. As we show, such structures can be realized with as-synthesized MOF particles in a single step.

**Fig. 3:**
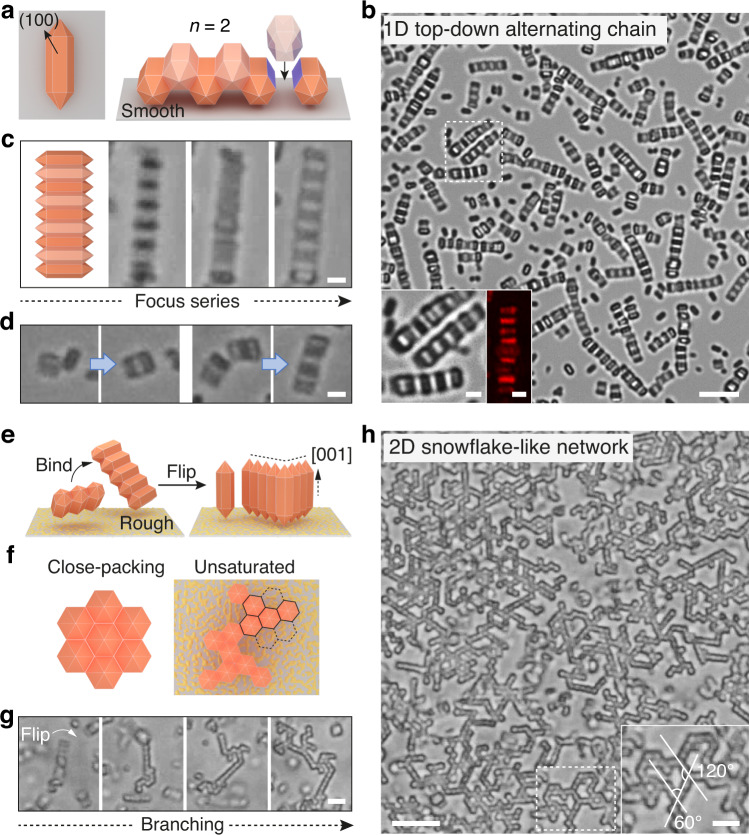
Self-assembly of MIL-88A particles. **a**–**d** 1D top-down alternating chain of MIL-88A particles formed on a smooth substrate. **a** Cartoon (left) shows the particle orients its (100) face towards the substrate by depletion attraction. Cartoon (right) shows the particles in the upper layer (light brick red) bind with those at the bottom (dark brick red) by the side faces (purple, *n* = 2). **b** Optical image of MIL-88A chains. Inset is the zoomed-in optical and reflected-light confocal image of the chains, both showing an alternating pattern. Optical images and cartoon in (**c**) show a microscope focus series of the chain. Snapshots from a movie (**d**) show the chain formation process. **e**–**h** 2D snowflake-like network of MIL-88A particles, formed on a rough substrate (gray plate with yellow dots). Schematic in (**e**) shows two short chains bind via (100) faces to form a branch, which flips over and stands on the substrate by their pyramidal tips ([001] direction). Cartoons in (**f**) compare the fully coordinated structure (*n* = 6, left) with the actual unsaturated network structure (*n* ≤ 6, right). Optical images in (**g**) show the flipping and branching process. Optical image in (**h**) shows the snowflake-like networks and the angles of branch junctions (inset). Scale bars: 5 and 1 μm (inset) for (**b**), 1 μm for (**c**, **d**), 2 μm for (**g**), 5 and 2 μm (inset) for (**h**).

One example is MIL-88A, a hexagonal framework featuring trimers of octahedral iron (III) units connected by fumarate dianions^41^. MIL-88A microcrystals are hexagonal rods with pyramidal tips (Fig. 3a), the assembly of which yields unexpected 1D and 2D superstructures. Specifically, when a smooth substrate is used, the settling particles have an affinity to it by their rectangular (100) faces; the triangular (101) faces are too small to establish effective contact. Unlike ZIF-8 RD/TRD particles, the substrate-bound MIL-88A particles exhibit no suitable faces within the plane that can bind. Instead, some particles slightly lift to the upper layer and bind with those on the substrate by contacting their side faces, resulting in linear chains with a top-down alternating configuration. The superstructure is observable by optical contrast under a bright-field microscope (bright/dark patterns) or a reflected-light confocal microscope (strong/weak reflection patterns), as shown in Fig. 3a, b. A microscope focus series also confirm the chain’s two-layer configuration (Fig. 3c and Supplementary Movie 5). Snapshots of a movie show the assembly process, where one rod binds with a dimer, followed by combining with another trimer to form a six-particle chain (Fig. 3d and Supplementary Movie 6).

When substrate confinement is eliminated by using a rough substrate, the same MIL-88A particles initially assemble by overlapping its (100) faces to form short straight chains suspending in solution (Fig. 3e). As each particle has six (100) faces available for binding, branches can develop when a particle in a chain binds more than two particles (Fig. 3f). The resulting branched assemblies then flip/reorient by gravity and settle onto the substrate, standing on their pyramidal tips. The reorientation lowers the mass center of the large assemblies. For a particle, the (100) faces are mostly not saturated in binding due to limited particle number around a branch point, shown in Fig. 3e-f by cartoon and Fig. 3g by optical microscopy. The process is also followed in Supplementary Movie 7. After incubation, instead of close-packing, 2D porous networks have formed; the branch junctions of the network adopt angles of 60˚ or 120˚, having a snowflake-like appearance, as shown in Fig. 3h.

### Anisotropic and directional assembly of UiO-66 particles

**Fig. 4:**
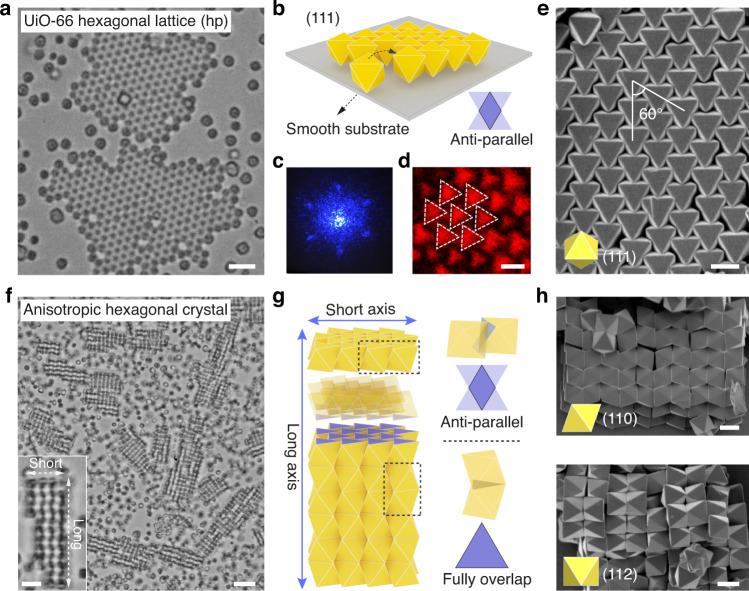
Anisotropic and directional assembly of UiO-66 particles. **a**–**e** 2D hexagonal superlattice (hp) assembled on a smooth substrate. Optical microscope image of the UiO-66 films is shown in (**a**). Cartoon in (**b**) shows particles sitting on the substrate by the triangular (111) face within the assemblies. They contact via their faces in an antiparallel fashion (shown in purple). Laser diffraction pattern of the resulting hexagonal lattice **c**. The (111) facet arrays are highlighted by reflected-light confocal microscopy (**d**) and SEM (**e**). **f**–**h**, Anisotropic quasi-1D stripe-like superstructure assembled from UiO-66 octahedra on a rough substrate. Optical image **f** shows the supercrystals; inset shows a crystal stripe and its long and short axes. Cartoons in (**g**) highlight the interparticle bonding within (along the short axis) and between hexagonal layers (along the long axis), featuring antiparallel (top right) and full facet overlap (bottom right), respectively. SEM images in (**h**) show the (110)- and (112)-oriented UiO-66 superstructures. Scale bars: 3 μm (**a**), 1 μm (**d**, **e**, **h**), 5 μm (**f**), and 2 μm (**f**, inset).

The colloidal assembly of UiO-66, a zirconium-carboxylate framework, is next explored. UiO-66 particles are highly symmetric octahedra with eight identical triangular (111) facets^42^. Upon assembly on a smooth surface, they form 2D films of a hexagonal lattice, where all particles orient their (111) facets to the substrate and bind with one another by overlapping the side facets in an antiparallel manner (Fig. 4a, b, Supplementary Fig. 11 and Movie 8). The symmetry of the superstructures is revealed by a home-made laser diffraction setup, shown in Fig. 4c and Supplementary Fig. 12. Reflected-light confocal and electron microscope images in Fig. 4d, e show that all the particles pack with an aligned orientation.

Surprisingly, when a rough substrate is employed, an elongated, stripe-like quasi-1D superstructure is formed (Fig. 4f and Supplementary Figs. 13–14). The anisotropic assembly is unexpected as the particle itself is highly symmetric and the substrate influence is absent. A close investigation by SEM reveals that the superstructure is composed of layers of hexagonally packed particles that further stack together (Fig. 4g, h). The particle facet partially contacts within each layer along the short axis in an antiparallel manner, while full facet overlaps are established between the layers along the long axis. These distinct modes of facet overlap, associated with unequal strength of depletion forces, manifest as directional crystal growth (Fig. 4g). The growth kinetics are observed in situ under a microscope, shown in Supplementary Fig. 14 and Movie 9. It reveals that the full facet overlap (or strong binding) facilitates the crystal growth along the long axis. Such a directional growth has not been observed in other colloidal systems including octahedral nanoparticles.

### 2D films of MIL-96 particles and truncation-dependent superstructures

**Fig. 5:**
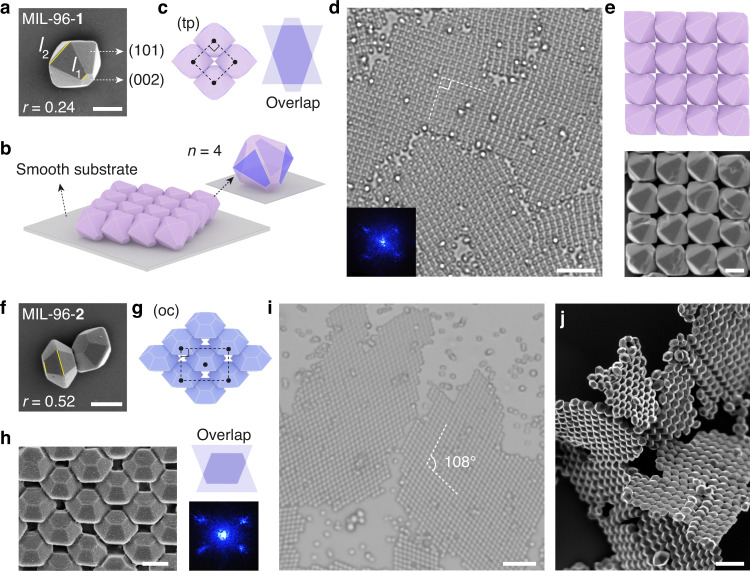
Truncation-dependent assembly of MIL-96 2D films. **a**–**e** Square (tp) lattice formed by MIL-96-**1**. SEM image (**a**) of MIL-96-**1** with small truncation (*r* = *l*_1_/*l*_2_ = 0.24); the (101) and (002) facets are labeled. Cartoon in **b** shows each MIL-96-**1** particle adheres to the substrate by (101) facet and packs with four neighboring particles by contacting their trapezoidal faces (purple) (*n* = 4). Cartoons in **c** show the unit cell and the antiparallel face overlap. Optical microscope image (**d**), laser diffraction pattern (**d**, inset), cartoon and SEM image (**e**) show the square lattices assembled from MIL-96-**1**. **f**–**j** Centered rectangular (oc) lattice formed by MIL-96-**2**. SEM image of MIL-96-**2** (*r* = 0.52) is shown in **f**. Cartoons (**g**), SEM (**h**), and optical microscope image (**i**) show the structure of MIL-96-**2** superlattice (axial angle = 108°). The unit cell is labeled in **g**. The face overlap and laser diffraction pattern are shown in (**h**). SEM image in **j** shows the pieces of MIL-96-**2** films. Scale bars: 0.5 μm (**a**, **e**, **f**, **h**), 5 μm (**d**, **i**), and 2 μm (**j**).

For MOF particles, truncation in shape can be easily obtained via synthesis, which provides an effective handle to tune the assembly. We show such effect with MIL-96, an aluminum-carboxylate framework belonging to the hexagonal crystal family^31,43^. The microcrystals adopt a unique truncated hexagonal bipyramidal shape possessing twelve trapezoidal (101) facets and two hexagonal (002) facets^44^ (Figs. 1a and 5). The ratio between the top and the bottom base of the (101) trapezoid, *r* = *l*_1_/*l*_2_, represents the degree of truncation.

MIL-96-**1** particles have a small truncation (*r* = 0.24, Fig. 5a). They assemble into a square lattice on smooth substrate, as shown in optical image and revealed by laser diffraction pattern in Fig. 5d. This result has not been anticipated because the particles possess a hexagonal symmetry. Cartoons in Fig. 5b, c unveil the particle orientation and packing within the superlattice. The particles are confined on the substrate by one of its trapezoidal (101) faces, while contacting four neighboring particles by overlapping other corresponding (101) faces (highlighted in Fig. 5b). The arrangement of particles is further revealed by SEM, showing ordered arrays (viewing from the top) that match the cartoon model (Fig. 5e). We note that the trapezoidal faces from bound particles adopt an antiparallel configuration. The pattern of facet overlap is shown in Fig. 5c, which, as we discuss below, is crucial in determining the lattice structures.

For MIL-96-**2** particles, which have a larger truncation (*r* = 0.52, Fig. 5f), they instead self-assemble into centered rectangular lattices with an axial angle of 108° (Fig. 5f–j, Supplementary Movie 10). Yet, like MIL-96-**1**, it is still the trapezoidal (101) facet that forms the bonds between the particles and with the substrate. The particle orientation and packing are illustrated in Fig. 5g and confirmed by SEM, laser diffraction and optical microscopy (Fig. 5h, i). In addition, it is possible to recover the 2D superlattices as films by lyophilization while the particle arrangement is preserved (Fig. 5j), which paves the way for their future utilization (e.g., device fabrication).

In these two cases, the effect of particle truncation on the assemblies can be understood by considering the overlap area of the trapezoidal faces. When *r* = 0.24, the trapezoids have an overlap around 71% within the square lattice (Fig. 5c). When *r* = 0.52, the same square lattice would give rise to a relatively small facet overlap (42%) and experience much steric hindrance (Supplementary Fig. 15). The particles need to slide within the substrate plane to increase the overlap area to 61%, resulting in the observed lattices in Fig. 5g. We note that, in both cases, the [002] direction of the particles are mutually oriented and is non-vertical to the substrate. Such particle arrangement is essential to their functions, as we discuss below.

### Birefringent crystalline MOF films by assembly

Having showcased the various MOF superlattices, we aim to couple this capability with the molecular structure and function of MOFs to create hierarchical materials with emerging properties. We approach this idea by considering the anisotropic properties of the crystalline frameworks, which are originally confined within individual MOF crystallite but are now mutually coordinated and extended over larger length scales. In this case, we demonstrate optical anisotropy of 2D films of MIL-96 particles.

**Fig. 6:**
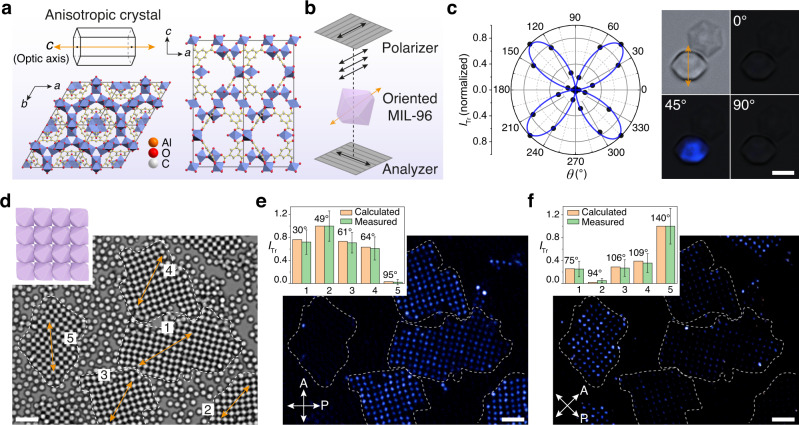
Birefringent 2D films of anisotropic MIL-96. **a** Molecular structure of MIL-96 along *c* and *b* axes (*a-b* plane and *a-c* plane). The optic axis (*c* axis) is shown by orange double arrow. Orange, red, and gray spheres represent aluminum, oxygen, and carbon atoms, respectively. Al octahedra are shown in blue. **b** Observation of birefringence using polarized light microscopy with crossed polarizer/analyzer (gray grating). The black double arrows show the polarization direction after light passes through the polarizer or analyzer. The transmission light intensity (*I*_Tr_) is recorded every 15° by rotating the crossed polarizer/analyzer pair. **c** Plot with fitting of the normalized *I*_Tr_ as a function of *θ*, angle between light polarization and MOF’s optic axis (projected on *x*–*y* plane). Bright-field and polarized optical images of 5.1-µm MIL-96 particles at *θ* = 0, 45, and 90°. MIL-96 particle standing by its (101) face exhibits typical birefringence of a uniaxial crystal; the one standing by the (002) face is always dark. **d** Bright-field optical image and cartoon of MIL-96 films assembled from 1.8-µm particles. The superlattice grains are circled by dotted line and labeled by their orientation (orange double arrows). **e**, **f** Crossed-polarized microscope images show birefringence of the superlattice grains in (**d**) at polarization angles of 0° (**e**) and 45° (**f**). Angle of each crystal grain with respect to polarization direction is measured and the theoretical intensities are calculated, which agree with the measured *I*_Tr_ (normalized) values (charts, insets). Scale bars: 3 µm (**c**) and 5 µm (**d**–**f**).

For MIL-96 framework, the *a-b* plane features hexagonal networks of aluminum octahedra, while the *a-c* (or *b-c*) planes are of a low symmetry, having connected sinusoidal chains of aluminum octahedra^43^ (Fig. 6a). As such, MIL-96 is an anisotropic uniaxial crystal (*c* is the optic axis), potentially useful for building birefringent material by our assembly scheme. The birefringence of individual MIL-96 microcrystal is first investigated, using polarized light microscopy equipped with crossed polarizers (Fig. 6b). When the particles sit on its (002) facet, the *c* axis is parallel to the incident light path, and they always appear dark as transmission of polarized light is prohibited. When the particle sits on its (101) facet, it exhibits anisotropic light transmission, depending on *θ*, angle between the direction of light polarization and the crystal’s *c* axis (projected on *x–y* plane). The light intensity reaches the maximum (particle appear bright) at *θ* = 45˚, and the minimum (particle appear dark) at *θ* = 0˚ and 90˚. The intensity follows *I*_Tr_ ~ *sin*^2^(2*θ*), as shown in Fig. 6c.

When the particles are assembled into 2D superlattices, the particles all sit by the (101) faces and with their optic axes mutually oriented. They establish coordinated optical anisotropy in 2D creating birefringent crystalline films. Figure 6d shows a bright-field image of 2D MIL-96 films consisting of multiple superlattice grains (circled by dashed lines and labeled as **1-5**) with different orientations by their *c* axis. Under polarized optical microscopy, these films showing orientation-dependent light transmission and the intensity matches the calculated values (Fig. 6e, f). When the polarization direction changes by 45˚, the films show flipped intensity, whereby the bright grains become dark and vice versa.

### Micropore alignment in superstructures enables fluorescence anisotropy

**Fig. 7:**
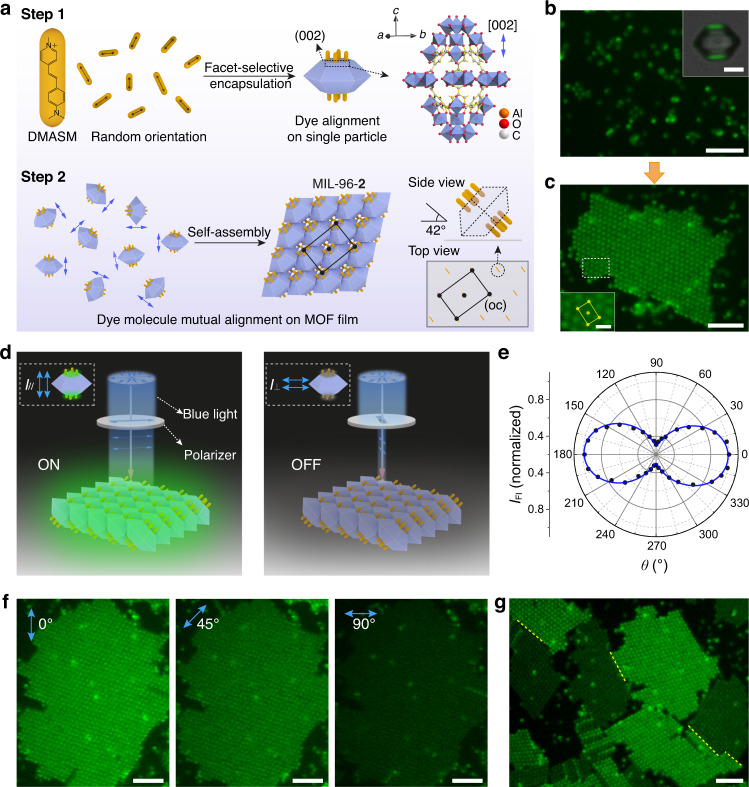
MOF films with anisotropic fluorescence and controlled micropore alignment. **a** Schematics showing DMASM dye molecules (orange rods) selectively recruited on (002) facets of MIL-96 particles (Step 1). The molecular structure of the dye is shown, and its transition dipole moment is labeled with black double arrow. The randomly oriented dye molecules in solution are encapsulated in the ellipsoidal pores of MIL-96 (structure shown) and their transition dipole moments are aligned to the pore (the [002] direction, purple double arrow). The dye-encapsulated particles (MIL-96-**2**) with random orientations are assembled to form MOF films with mutually oriented dye arrays (Step 2). The side view of dye orientation (angle with respect to substrate is 42°) and their spatial arrangement in a centered rectangular lattice are shown. **b**, **c** Fluorescence microscope images of dye-encapsulated MIL-96-**2** particles before (**b**) and after (**c**) self-assembly. Inset (**b**) shows a large MIL-96 particle with fluorescence located at the (002) faces; inset **c** is the zoomed-in MOF film. **d** Illustration of the angle-dependent emission of MIL-96-**2** films excited by linearly polarized light (blue double arrows). The direction of polarization is parallel (left) and perpendicular (right) to the dye orientation to turn on and off the emission. **e** Azimuthal plot of the fluorescence intensities (*I*_Fl_) of MIL-96-**2** films as a function of *θ*, angle between polarization direction and dye orientation (also *c* axis of MIL-96). **f** Representative fluorescence images show strong, intermediate, and weak fluorescence of the MIL-96-**2** film in response to the polarized light at *θ* of 0°, 45° and 90°. **g** Fluorescence image displays grains of MIL-96-**2** film with diverse orientations and their boundaries (yellow dotted lines). Scale bars: 5 μm (**b**, **c**, **f**, **g**), 2 μm (**b**, inset), and 1 μm (**c**, inset).

The MIL-96 framework also possesses ellipsoidal micropores along the *c* axis^43^, which may afford directional entrapment of guest molecules. Indeed, we show that a rod-shaped dye, 4-[*p*-(dimethylamino)styryl]−1-methylpyridinium (DMASM)^45^, can be adsorbed by MIL-96 particles and locate preferentially at the particle’s (002) facets (Fig. 7a, b, inset). The dye molecules enter the micropores via the (002) facets but further diffusion to the particle interior is limited by the narrow inter-pore passages^46^. Nevertheless, the dyes are directionally entrapped, with their long axes aligned to the *c* axis of MIL-96, as confirmed by experiments described in Supplementary Fig. 16 and Movie 11.

The dye-containing particles, in this case MIL-96-**2**, are then assembled to form 2D films (centered rectangular lattice) (Fig. 7a–c). As the particles are mutually oriented, the micropores and the encapsulated dye molecules are synchronized and aligned across the film. When excited with linearly polarized blue light (470 nm), the film shows anisotropic fluorescence, with its intensity dependent on the directions of light polarization. Strong fluorescence is observed when the polarization is parallel to the *c* axis of the particles (its projection on *x–y* plane; the angle between *c* axis and the substrate is about 42˚), which aligns with the dye’s transition dipole moment. When the light is perpendicular, the fluorescence is turned off, as illustrated in Fig. 7d. Optical images in Fig. 7f show the fluorescence of a film when the polarizations are 0°, 45°, and 90°, respectively. The angular dependence is further recorded and plot as a function of polarization direction, following the expected *I*_Fl_ ~ cos^2^(*θ*) relationship^47^ (Fig. 7e, see also Supplementary Fig. 17 and Movie 12). Moreover, the fluorescence anisotropy can image multi-grain crystalline films and defects, revealing grain-dependent fluorescence and grain boundaries (Fig. 7g and Supplementary Movie 13).

While rationally designed films with optical anisotropy are fundamental for optical components^48^, the self-assembly of MOF particles, when coupled with molecular structures and functions, provides a simple and more flexible method.

## Discussion

By utilizing depletion interaction and controlling the particle orientation via substrate modification, our technique exploits the polyhedral shapes of common MOF particles of colloidal sizes and enables highly directional colloidal bonding, giving rise to a wide range of assemblies. We have further shown the mutual alignment of the MOF microstructures and the coordination of their functions, allowing us to create hierarchical materials such as films with anisotropic properties.

In what follows, defect-free films of large area can be fabricated with the aid of, for example, seeding or external field^26^, for practical applications. Our method is advantageous as the film should in principle grow on various surfaces (e.g., electrodes) regardless of their composition. No crystal lattice matching is need, as required in the films produced by epitaxial growth^15,49,50^. Given the facts that more than 90,000 different types of frameworks^51^ have been developed and that our strategy is universally applicable, our next steps are to explore how the coordinated microstructure and superstructure can be coupled with diverse other functions of MOFs, including specially designed varieties. We envisage the emergence of numerous advanced materials that are otherwise inaccessible, with importance towards electrochemistry, catalysis, and separation, to name a few.

Lastly, with the large pool of MOFs available, our strategy offers exciting opportunities to the field of colloidal chemistry, where one of the foci is to synthesize anisotropic particles to build exotic structures^10–12^. The properties of particles (e.g., index of refraction for photonic applications) are not limited to those of polystyrene, silica, and titania, but are easily modified via MOF design, or simply by adsorption of guest molecules or encapsulation of nanoparticles^52,53^.

## Methods

### Synthesis of micrometer-sized polyhedral MOF colloids

MOF microcrystals of different types were synthesized under various solvent/temperature conditions by mixing metal source, ligands, and modulators. ZIF-8 particles were synthesized in purely aqueous or mixed solvent conditions^21,35^. Those with a rhombic dodecahedron (RD) shape were made in methanol and water (volume ratio, 1:1), using Zn(NO_3_)_2_·6H_2_O as metal source and poly(vinyl pyrrolidone)/1-methylimidazole as modulators. RD or TRD ZIF-8 particles with smaller sizes were obtained in purely aqueous condition, using Zn(OAc)_2_·2H_2_O as metal source. Cetyltrimethylammonium bromide (CTAB) was used as the modulator for the synthesis of cubic ZIF-8 particles with truncations. MIL-88A particles were synthesized using a modified hydrothermal route^54^. Fe(III) salt and fumaric acid were the metal source and linkers, while Pluronic F127 and acetic acid (HAc) were the modulators to control the particle size and morphology. UiO-66 and MIL-96 particles were synthesized following solvothermal methods^42,44^. The synthetic conditions were adjusted to obtain MIL-96 particles with different truncations, MIL-96-**1**, -**2**, and large particles 4.5 and 5.1 µm in sizes.

### Self-assembly of MOF colloids by depletion interaction

#### Stock solution preparation

The MOF particles were dispersed in stock solutions containing a certain amount of cetyltrimethylammonium chloride (CTAC) (for all particles except for MIL-88A) or sodium dodecyl sulfate (SDS)^55^ (for MIL-88A). The MOF colloidal suspension was washed at least three times by centrifugation and redispersion in DI water. In the final cycle, the particles were dispersed in aqueous CTAC 10 mM or SDS 50 mM in concentration.

#### Self-assembly

To obtain the appropriate depletant and particle concentration for self-assembly, the stock solution was first diluted by adding a certain amount of DI water (to reach the target depletant concentration). The particles were then centrifuged, and a fraction of the supernatant was removed to concentrate the particle accordingly. Supplementary Table 1 shows the detailed experimental conditions for assembling colloidal MOFs with various kinds, sizes, and shapes. The suspension was charged and sealed in a rectangular glass capillary tube (20 × 0.10 × 2.00 mm) for self-assembly. The capillary tube also provides a flat substrate that guides the assembly.

#### Substrate modification

To eliminate the depletion attraction between particles and substrate, the glass capillary tube was modified to bear a rough surface^56^ (Supplementary Fig. 3). The capillary tube was silanized by 1.0% w/w (3-aminopropyl)triethoxysilane (APTES) in absolute ethanol solution at room temperature for 24 h. Then, the silanized tube was immersed in a suspension of poly(NIPAM-*co*-AAC) hydrogel particles (*d* = 285 ± 14 nm by DLS) before use.

### Light diffraction

The MOF superlattices were characterized by light diffraction using a home-built apparatus (Supplementary Fig. 12). A blue violet laser (*λ* = 405 nm, 13% of 4.5 mW, output from an optical fiber) was fixed to optical table. The capillary tube with MOF superstructures was attached to a glass slide, which was mounted to a holder. The direction of the incident laser was set to perpendicularly transmit the bottom of the capillary tube. A white reflector board, to which the diffraction patterns were projected, was placed some distance from the sample. The distance was adjusted from 6.0 to 9.0 cm, depending on lattice parameters of the assemblies from various kinds of MOFs. The diffraction patterns were recorded using a digital camera.

### Fixing the assemblies via lyophilization

To fix the superstructures assembled from MOF particles for SEM imaging, the samples in the capillary tubes were lyophilized. Then, the capillary tube was slit and the glass pieces with MOF assemblies were placed on the SEM stub for further imaging.

### Birefringence measurement

Samples of MIL-96 particles or assembled films were mounted on the microscope stage. The microscope is equipped with crossed polarizers (i.e., one polarizer and one analyzer) along the path of the transmission light. The sample is located between the two polarizers. The crossed polarizers are simultaneously rotated with respect to the sample. The angle *θ* describes the rotation angle between the polarization direction of the incident light and the optic axis of the crystal (projected on *x–y* plane). The transmitted light signal dependent on *θ* was captured by a CCD camera. The experiment was done on a Nikon Eclipse Ti-2 inverted microscope. The angle measurement error is ±1°.

### Fluorescence anisotropy of the MIL-96 2D superlattice

#### Facet-selective encapsulation of dye molecules on individual particles

The cationic dye, 4-[*p*-(dimethylamino)styryl]-1-methylpyridinium (DMASM), was selectively encapsulated on the (002) facets of the MIL-96 particles (MIL-96-**2** and 4.5-µm particles were used). Specifically, MIL-96 particles were suspended in aqueous solution containing DMASM (0.5 mg/ml). After 2 h, the particles were centrifuged, and the supernatant was removed. MIL-96-**2** particles were used for assembling and 4.5-µm particles were re-dispersed in DI water for directly observation using a Leica optical/fluorescence microscope equipped with a polarizer.

#### Self-assembly of dye-encapsulated MIL-96 colloidal superlattices

The above dye-encapsulated MIL-96-**2** particles were then re-dispersed in 0.5 ml of water containing a mixture of CATC (10 mM) and DMASM (0.05 mg/ml) to make a stock solution. A certain amount of DMASM in solution was needed to maintain the absorption equilibrium on MOFs particles. The above suspension containing MIL-96-**2** was diluted to the appropriate concentrations (CTAC: 4.4 mM) and charged in pristine glass capillary tube for assembly. The sample with more defects was prepared when the CTAC concentration was 5.15 mM.

#### Fluorescence anisotropy

The samples of DMASM-encapsulated 4.5-µm MIL-96 single particles or MIL-96-**2** films was then mounted on the stage and illuminated using linearly polarized light. The filtered excitation light (470 ± 25 nm) from a LED light source (pE-300lite) was first allowed to pass a polarizer before hitting on the sample. The polarizer can be rotated with a constant speed to alter the direction of light polarization from 0° to 360°. The fluorescence signal passing through an emission filter (525 ± 25 nm) was captured by CCD detector. The experiment is done on a Leica DM2000 upright microscope.

#### Data treatment

The sequence of fluorescent images was analyzed using ImageJ, which reads the mean fluorescence intensities of the particle assemblies as well as the background. The intensities of the background were extracted from those of the assemblies, which were then normalized. The relation between the normalized intensities and the polarization direction (in angle) was found as *I* = 0.68 cos^2^ *θ* + 0.3.

### Microscopy

#### General

The MOF particles and their assemblies were observed on a Nikon Eclipse Ti-2 inverted microscope equipped with a Nikon D7000 DSLR camera, or on a Leica DM2000 upright microscope with a MC170 camera. Fluorescent images were taken using a Leica SP8 lasing scanning confocal fluorescence microscope. The fluorescence anisotropy is observed on the Leica DM2000 microscope equipped with a polarizer. The particles in the dried state and the freeze-dried assemblies on the capillary tubes were imaged by a TESCAN MAIA3-XMH scanning electron microscope at low voltages (0.8–1.5 kV), using the in-beam secondary electron detector. A thin-layer (~5 nm) of gold was sputter-coated on the sample for the high-resolution imaging.

#### Reflected-light confocal microscopy

The orientation of MOF particles in the assemblies was investigated using a Leica SP8 confocal fluorescence microscope under a reflected-light mode. Briefly, the photomultiplier tube (PMT) was set to collect light the same wavelength of the incident laser. In this case, the signal collected is the reflected (scattered) light from the particle surfaces. This mode is very effective to observe the flat facets that face to the substrate, clearly showing their shapes. No fluorescent dyes are needed for labeling the particles.

#### Atomic force microscope (AFM) imaging

Surface roughness of pristine and modified glass substrates were characterized using a Bruker NanoWizard ULTRA Speed 2 AFM. For the modified rough substrate, the glass tube coated with poly(NIPAM-co-AAC) nanoparticles was dried and the hydrogel nanoparticles shrank. The images were captured in air with scan areas of 1.5 × 1.5 µm using quantitative imaging (QI) mode at a scan rate of 1 Hz. The tip used was SCM-PIT-V2 with a spring constant ~2.8 N/m. JPK Data Processing software was used to analyze the average roughness.

## Supplementary information

Peer Review File

Description of Additional Supplementary Information

Movie 1

Movie 2

Movie 3

Movie 4

Movie 5

Movie 6

Movie 7

Movie 8

Movie 9

Movie 10

Movie 11

Movie 12

Movie 13

Supplementary Information

## Data Availability

Raw data for all figures, plots, and particle size distributions are available from the corresponding author upon request.
