# Supplementary material for: Low-dimensional assemblies of metal-organic framework particles and mutually coordinated anisotropy
Source: Nat Commun. 2022 Jul 9;13:3980. doi: 10.1038/s41467-022-31651-3 (PMC9271095; doi:10.1038/s41467-022-31651-3)
Supplement: Supplementary file 2 — Description of Additional Supplementary Information [file 41467_2022_31651_MOESM2_ESM.pdf]

Supplementary Movie Legends 1-13

Supplementary Movie 1 | Self-assembly of RD ZIF-8 particles on a rough substrate. This movie shows the formation of quasi-3D/3D superstructures with RD ZIF-8 particles on a rough substrate. The nucleation and growth dynamics is shown in (part 1), and the focus series of the supercrystals are shown in (part 2). The movies are played 100× and 1× of real time, respectively.

Supplementary Movie 2 | Formation of 1D chains with truncated rhombic dodecahedral (TRD) ZIF-8 particles on a smooth substrate. Movie shows the binding between monomers and small oligomers of TRD ZIF-8 particles (highlighted with white circles), indicating a step growth kinetics. Movies with zoomed-in view (part 1) and large view (part 2) are shown; they are played 3× and 60× of real time, respectively.

Supplementary Movie 3 | Kinetics of self-assembly of truncated ZIF-8 cubes on a smooth substrate. This movie shows the self-assembly process of ZIF-8-**2** cubes with large truncation (r = 0.18). The nucleation and growth process of the square lattice is observed. The movie is played 40× of real time.

Supplementary Movie 4 | Effect of particle truncation on the self-assembly of ZIF-8 cubes. In this movie, the self-assembly of cubic ZIF-8 particles with different degrees of truncations are shown. (Part 1) The sliding between different rows of particles is obvious for ZIF-8-**1** cubes with small truncation and rounded edges (r = 0.08). (Part 2) No row sliding is observed for ZIF-8-**2** cubes with large truncation (r = 0.18). The movie is played 3× of real time.

Supplementary Movie 5 | Observation of the top-down alternating structure assembled

from MIL-88A particles. Focus series of one chain assembled by MIL-88A particles (on a smooth substrate) show the structure alternates in the *z* direction.

Supplementary Movie 6 | Formation of MIL-88A chains with top-down alternating configuration. This movie shows zoomed-in view (part 1) and large view (part 2) of the self-assembly process of MIL-88A particles utilizing a smooth substrate. The particles in the upper and bottom layers can be distinguished by the optical contrast and are represented by the cartoon in different colors. The monomers and small oligomers are highlighted using white circles. The movies are played 3×, 20×, 3×, and 60× of real time (in sequence).

Supplementary Movie 7 | Formation of 2D snowflake-like network with MIL-88A particles on a rough substrate. MIL-88A particles form short chains and develop branches to form snowflake-like superstructures. Movies with zoomed-in view (part 1) and large view (part 2) are shown; they are played 10× of real time.

Supplementary Movie 8 | Kinetics of self-assembly of UiO-66 particles on a smooth substrate. UiO-66 particles first form small nuclei, which continues to grow to form 2D hexagonal superlattices by incorporating more particles. The movie is played 40× of real time.

Supplementary Movie 9 | Directional growth of UiO-66 quasi-1D stripe-like supercrystals on a rough substrate. This movie shows the anisotropic self-assembly of UiO-66 particles due to two different patterns of face overlap. The movie is played 400× of real time.

Supplementary Movie 10 | Kinetics of self-assembly of MIL-96 particles. This movie shows the self-assembly process of MIL-96 particles with different degrees of truncations *r*. MIL-96-**1** particles (part 1, $r = 0.24$) and MIL-96-**2** particles (part 2, $r = 0.52$) form square lattice and

centered rectangular lattice on a smooth substrate, respectively. The movies are played 40× and 150× of real time, respectively.

Supplementary Movie 11 | Facet-selective encapsulation of DMASM and fluorescence anisotropy of single MIL-96 particles. 4.5-μm MIL-96 particles with encapsulated DMASM dye are shown. Fluorescence is only observed on the (002) facets of the particles when they are illuminated by linearly polarized light (blue arrow). This movie shows three particles with different orientations. White arrows in bright-filed microscope image (inset) indicate the orientation of dye molecules (0°, 45° and 90° for particle **1**, **2**, and **3**). By changing the direction of polarization from 0° to 360°, particles **1**, **2**, and **3** turn "on" and "off" sequentially, which reveals fluorescence anisotropy of individual particle.

Supplementary Movie 12 | Fluorescence anisotropy of dye-encapsulated MIL-96 colloidal superstructures. This movie shows the fluorescence of a large film, assembled from 0.8-μm MIL-96-**2** particles, in response to linearly polarized light. The dye orientation on the film is indicated by white arrow in the cartoon and the direction of light polarization (0-720°) is shown by the rotating blue arrow. The angle-dependent fluorescence of the film is recorded (inset, plot).

Supplementary Movie 13 | Dye-encapsulated MIL-96 colloidal superstructures: different crystal grains with grain boundaries. MIL-96-**2** films with multiple crystal grains with different directions (white arrows); they show fluorescence of different intensities at the same polarization direction. The grain boundaries (dashed boxes) are observed between adjacent crystal grains.
