# Supplementary material for: Low-dimensional assemblies of metal-organic framework particles and mutually coordinated anisotropy
Source: Nat Commun. 2022 Jul 9;13:3980. doi: 10.1038/s41467-022-31651-3 (PMC9271095; doi:10.1038/s41467-022-31651-3)
Supplement: Supplementary file 16 — Supplementary Information [file 41467_2022_31651_MOESM16_ESM.pdf]

Supplementary Information

Low-Dimensional Assemblies of Metal-Organic Framework Particles and Mutually Coordinated Anisotropy

Lyu *et al.*

Supplementary Figures

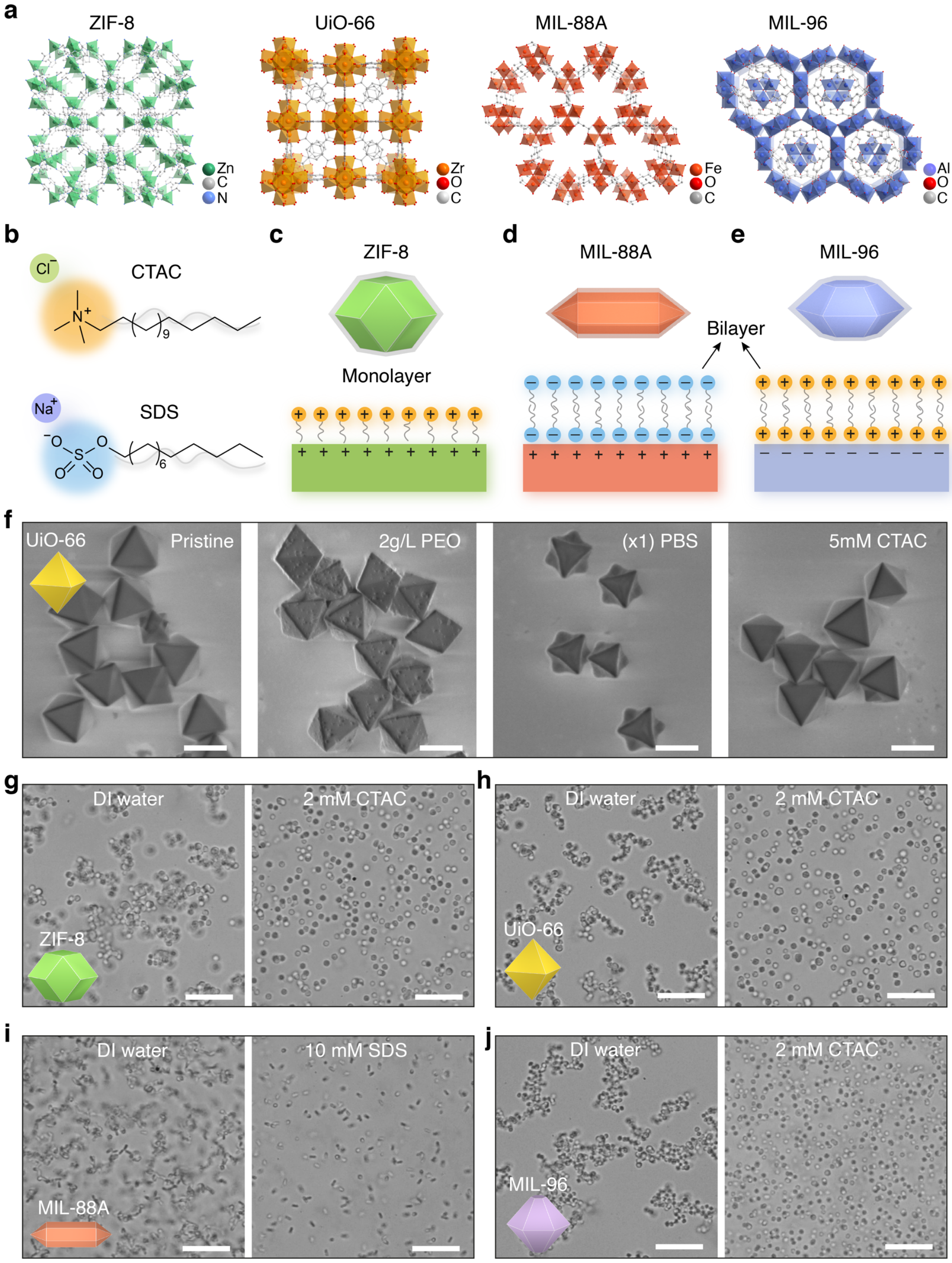

Supplementary Figure 1. MOF particles stabilized by ionic amphiphiles. a, Crystal structures of common MOFs used in this work. Atom color code: Zn, green; C, grey; N, blue; Zr, orange; O, red; Fe, brick red; Al, violet. **b**, Molecular structures of ionic amphiphiles (CTAC and SDS). **c-e**, Cartoons show ionic amphiphiles form a monolayer or bilayer on the surface of

MOF, depending on the surface charge. For example, CTAC in principle form a monolayer on ZIF-8 (**c**) and bilayer on MIL-96 (**e**); SDS form a bilayer on MIL-88A (**d**). The adsorption could be due to electrostatic or hydrophobic interaction. The zeta potentials of different MOF particles in water with/without ionic amphiphiles are provided in Supplementary Table 2. **f**, Scanning electron micrographs (SEMs) show pristine MOF particles (UiO-66 as an example) and their morphological changes when they are soaked in different aqueous solutions for 12 hours. From left to right: deionized (DI) water, 2.0 g/L poly(ethylene oxide) (PEO) (MW: 1 000 kDa), phosphate-buffered saline (PBS), and 5.0 mM CTAC. PEO polymers absorb unevenly on MOF surface; the particles are etched in PBS while they remain intact in CTAC solution. **g**-**j**, Optical microscope images show that ZIF-8 (**g**), UiO-66 (**h**), MIL-88A (**i**), and MIL-96 (**j**) particles form large aggregates after washing and dispersing in DI water (left column). When mixed with ionic surfactants whose concentrations (2 mM for CTAC and 10 mM for SDS) are above their critical micelle concentrations (CMC) but below ones needed for self-assembly, the MOF particles are well-dispersed (right column). Insets are the cartoons of different MOF particles. Scale bars: 1μm (**f**) and 10 μm (**g**-**j**).

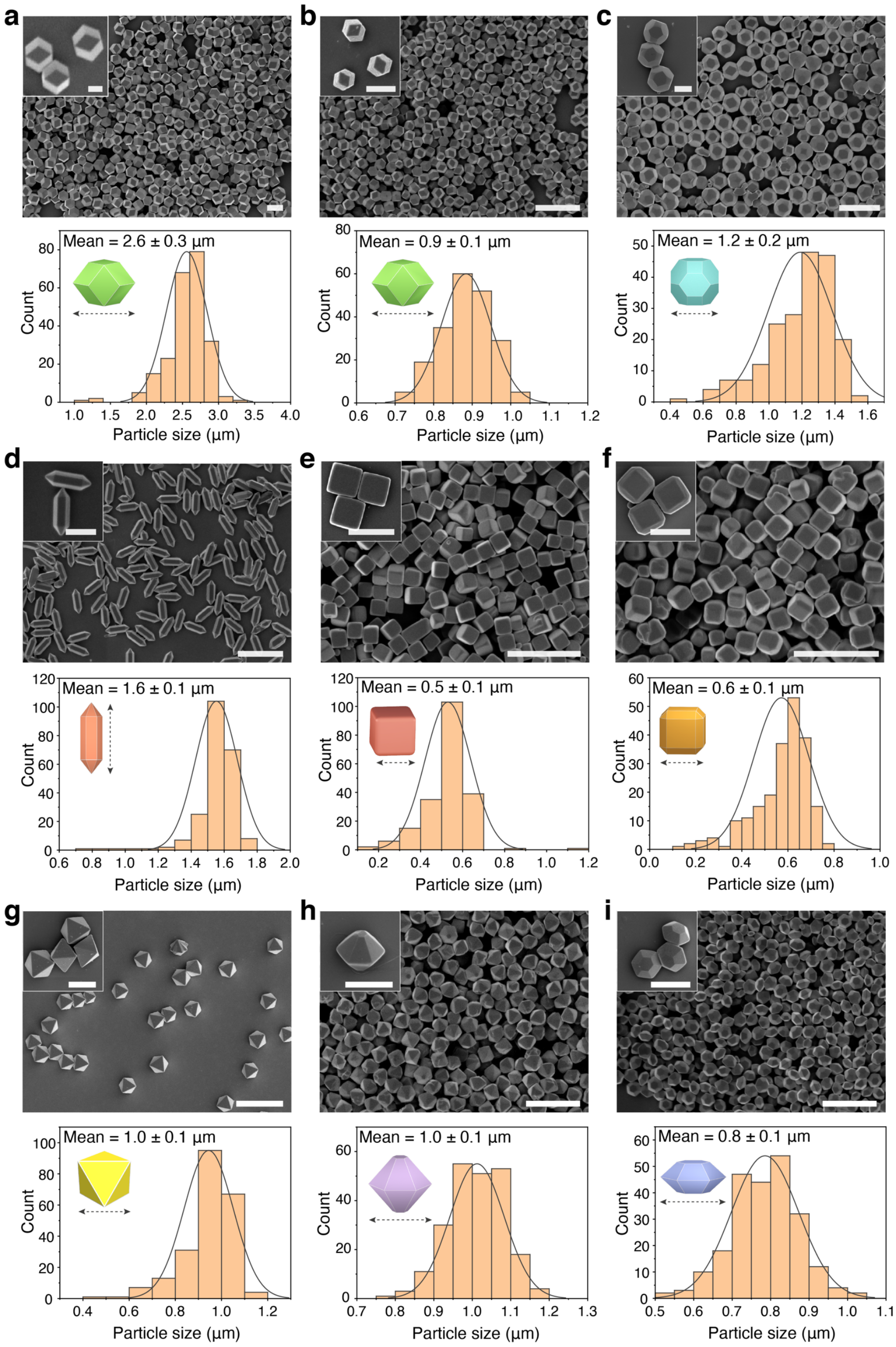

Supplementary Figure 2. MOF particles. Large view and zoomed-in SEMs of colloidal MOF particles with different sizes and shapes obtained from various MOF families (top panels). Their size distributions are shown in the bottom panels. The arrows below the cartoons (insets) show the measured particle dimensions. **a-b**, RD ZIF-8 particles of 2.6 ± 0.3 µm and 0.9 ± 0.1 µm. **c**, TRD ZIF-8 particles of 1.2 ± 0.2 µm. **d**, MIL-88A particles of 1.6 ± 0.1 µm. **e**, Cubic ZIF-8-**1** particles of 0.5 ± 0.1 µm. **f**, Cubic ZIF-8-**2** particles of 0.6 ± 0.1 µm. **g**, UiO-66 octahedra of

1.0 ± 0.1 μm. **h**, MIL-96-**1** of 1.0 ± 0.1 μm. **i**, MIL-96-**2** of 0.8 ± 0.1 μm. Scale bars: 3 μm and 1 μm (insets).

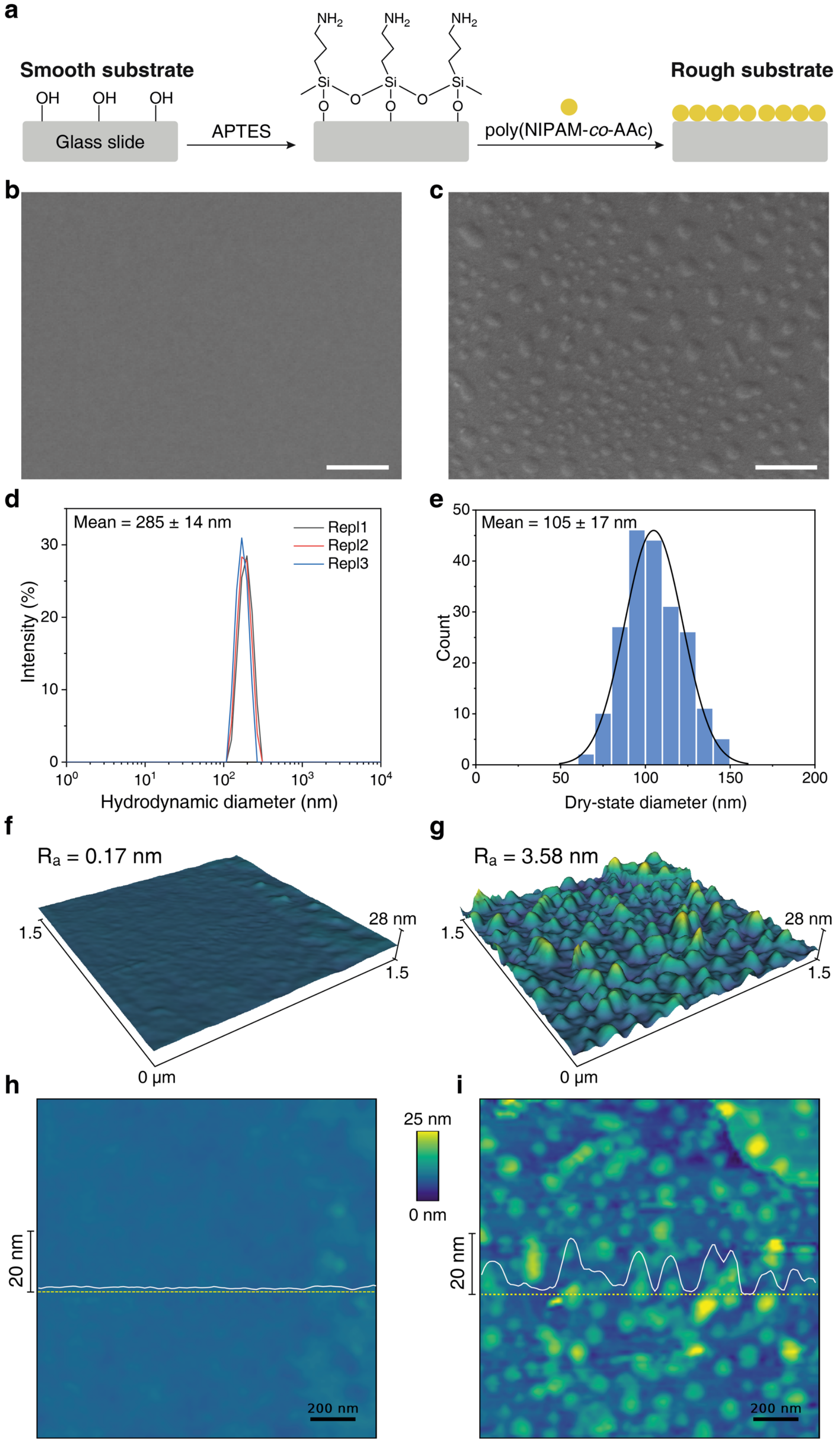

Supplementary Figure 3. Substrate modification. **a**, Schematics showing the process to modify the glass substrate to render it rough. Structure of functional group on the surface of glass slide (grey) are shown. Yellow spheres represent the poly(NIPAM-*co*-AAC) hydrogel nanoparticles. **b-c**, SEM images showing the smooth (**b**) and rough (**c**) substrates. **d**, Hydrodynamic diameter of poly(NIPAM-*co*-AAC) hydrogel nanoparticles measured by DLS. The measurement is repeated for three times. **e**, Size distribution of the poly(NIPAM-*co*-AAC) nanoparticles at dry state, measured from the SEM images. **f-i**, AFM images of pristine glass (**f**, **h**) and rough substrate (**g**, **i**). R_a represents average surface roughness in nanometers; AFM images in 3D and 2D views are shown; line scan profiles (white traces) and the cross section (yellow dotted lines) through the 2D images are shown. Scale bars: 0.5 μm (**b**, **c**) and 0.2 μm (**h**, **i**).

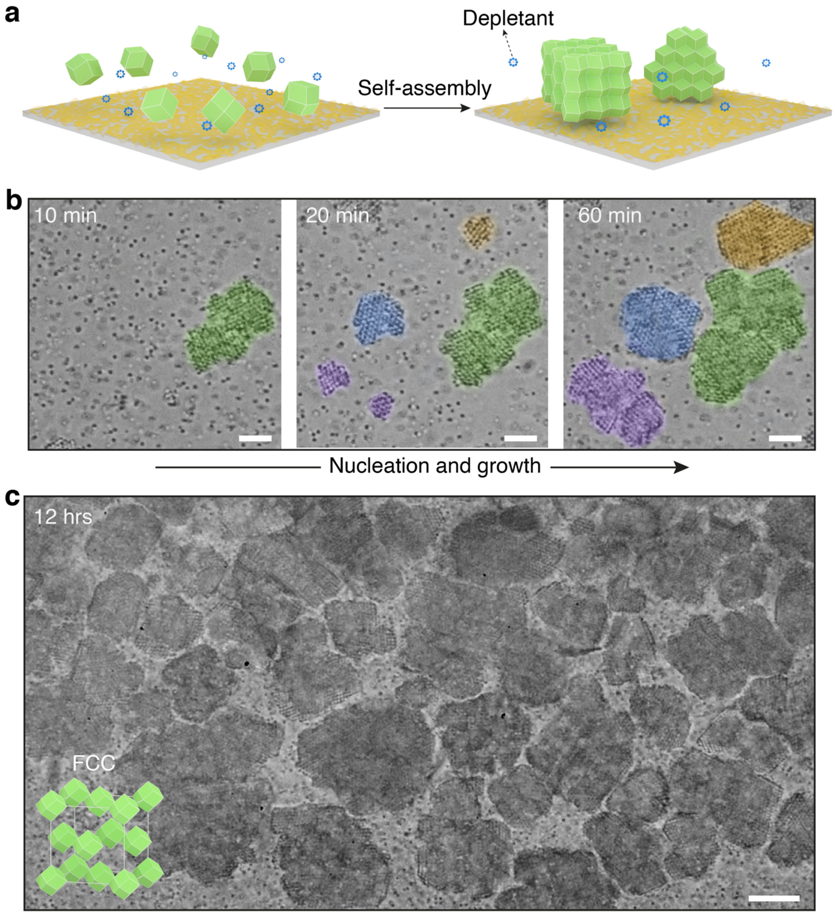

Supplementary Figure 4. Self-assembly of ZIF-8 particles on a rough substrate. a, Cartoons illustrating the self-assembly of ZIF-8 particles (RD, green) on the modified substrate (grey plate with yellow dots). Blue circles represent the CTAC depletants. **b**, Time-lapse microscope images showing the nucleation and growth of colloidal crystals of 0.9 µm-RD ZIF-8 particles. Different crystallites are indicated by colors. **c**, Bright-filed image of big chunks of quasi-3D/3D ZIF-8 supercrystals after 12 hours of assembly. The inset shows the face-centered cubic structure (FCC) assembled from the MOF particles. Scale bars: 5 µm (**b**) and 10 µm (**c**).

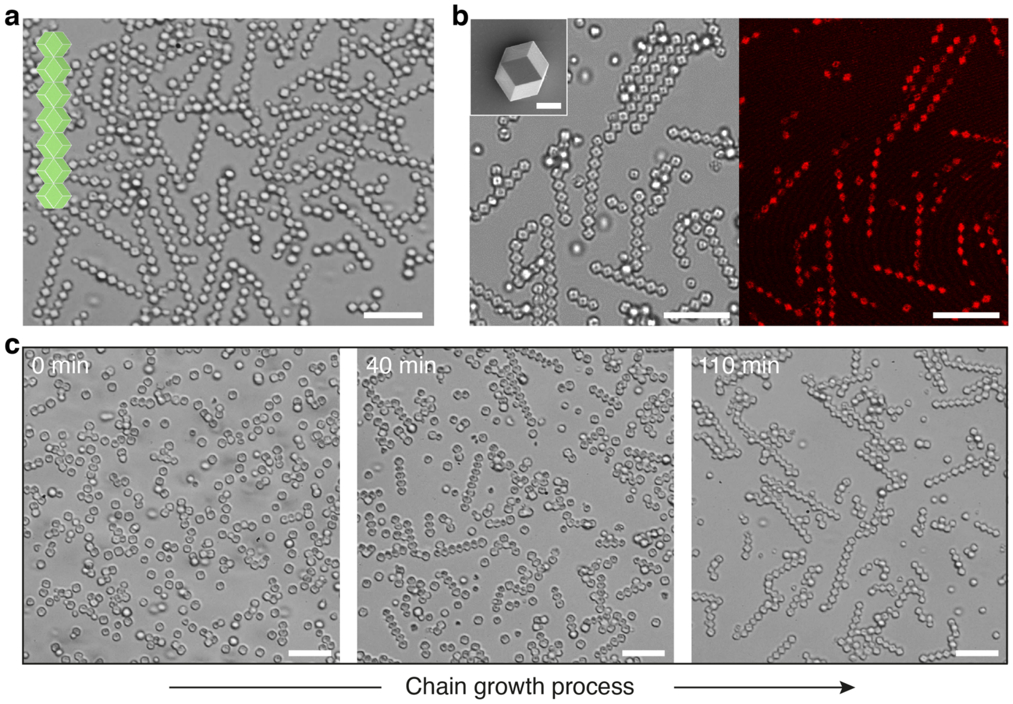

Supplementary Figure 5. 1D Chains assembled from ZIF-8 RD particles through substrate confinement. a-b, Cartoon, bright field and reflected-light confocal microscope images show the chain structure assembled from ZIF-8 RD particles (2.6 ± 0.3 μm) constrained on the smooth substrate. Inset of (**b**) is the SEM of the particle. **c**, Time-lapse microscope images show the self-assembly process, from single particles to long chains. scale bars: 10 μm and 1 μm (**b**, inset).

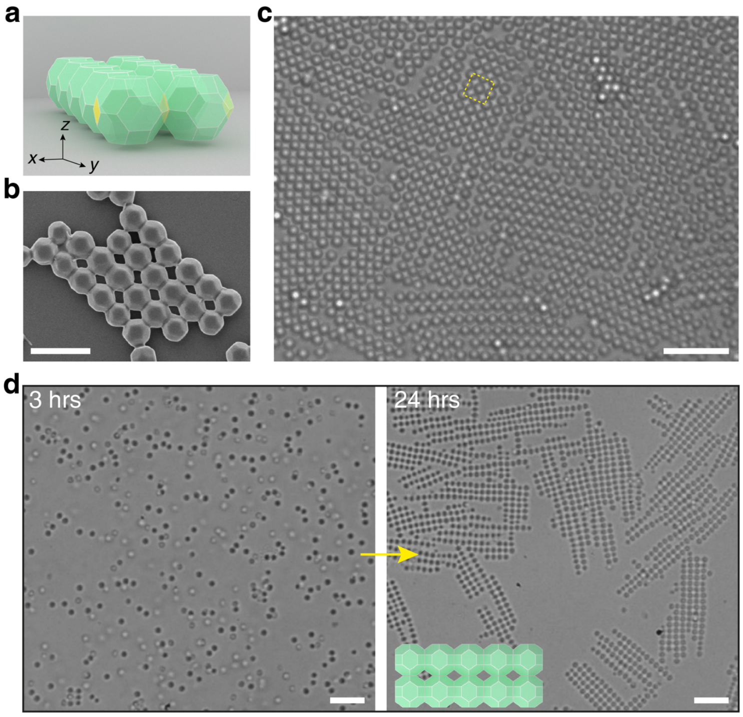

Supplementary Figure 6. Square lattices assembled from 0.8-μm TRD ZIF-8 particles. a, Cartoon illustrates that TRD ZIF-8 particles (aquamarine) can also bind by their square (100) facets (yellow) to form a square lattice. **b-c**, Representative SEM image (**b**) and a large view optical microscope image (**c**) show the structure of the resulting assemblies. **d**, Microscopies showing the self-assembly process. The particle number density is diluted by five times (~1000 per screen) and 5 mM CTAC is used for weaker depletion interaction. In this case, the kinetics of self-assembly is slow and there is barely binding in the first 3 hours (left). Almost all particles sediment to the bottom and the square lattice formed after 1 day. Cartoon in the inset shows the structure of the resulting assemblies. Scale bars: 2 μm (**b**) and 5 μm (**c-d**).

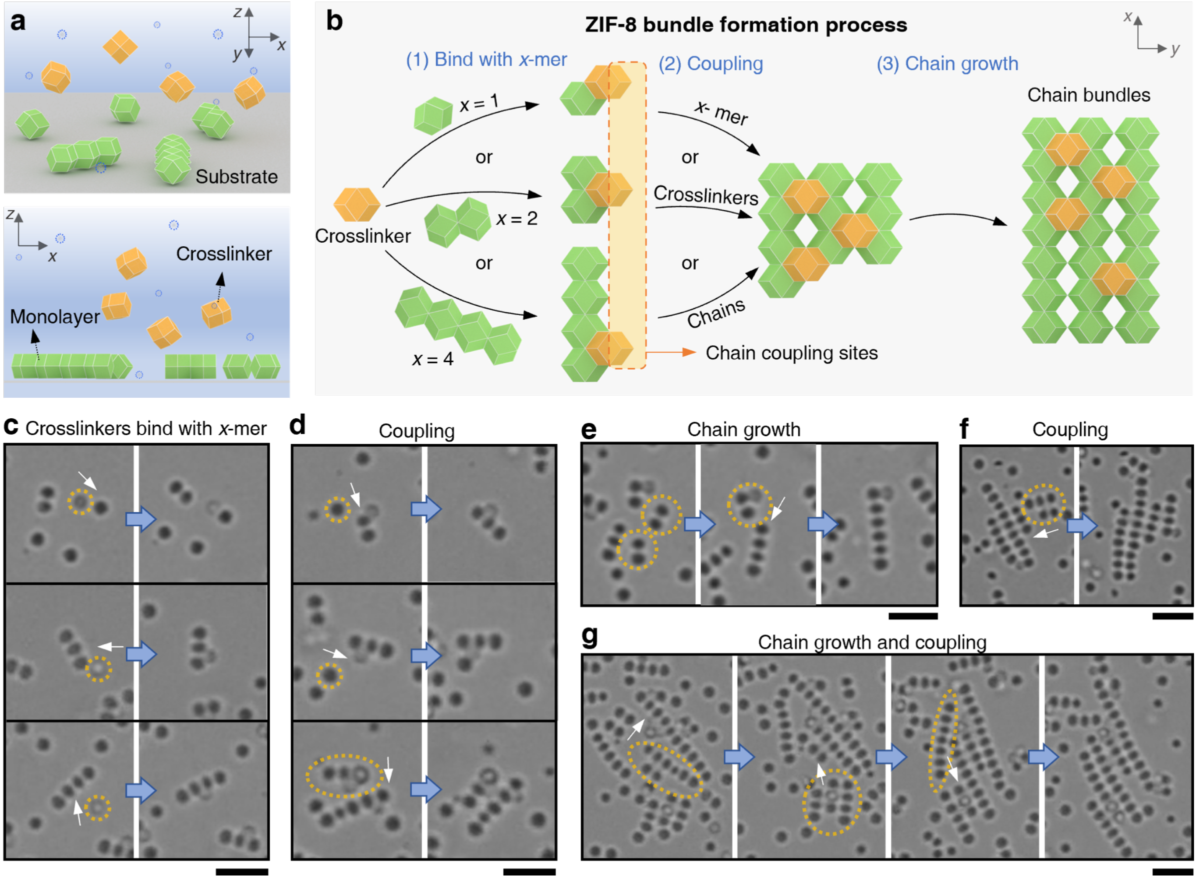

Supplementary Figure 7. Formation of ZIF-8 chain bundles. **a**, Perspective (top) and side view (bottom) of ZIF-8 particles during self-assembly on a smooth substrate (grey). The particles above the monolayer plane can be considered as “crosslinkers” (orange RD). **b-g**, Schematics (**b**) and representative optical microscope images (**c-g**) showing the formation process of ZIF-8 chain bundles. The crosslinkers can bind with *x*-mers (chain with *x* particles) on the monolayer plane to form clusters (**c**), which can couple with one another to form short bundles (**d**). The bundle structure is enlarged upon further chain growth and coupling (**e-g**). The particles during binding process are highlighted with orange circles in (**c-g**). Scale bars: 3 μm.

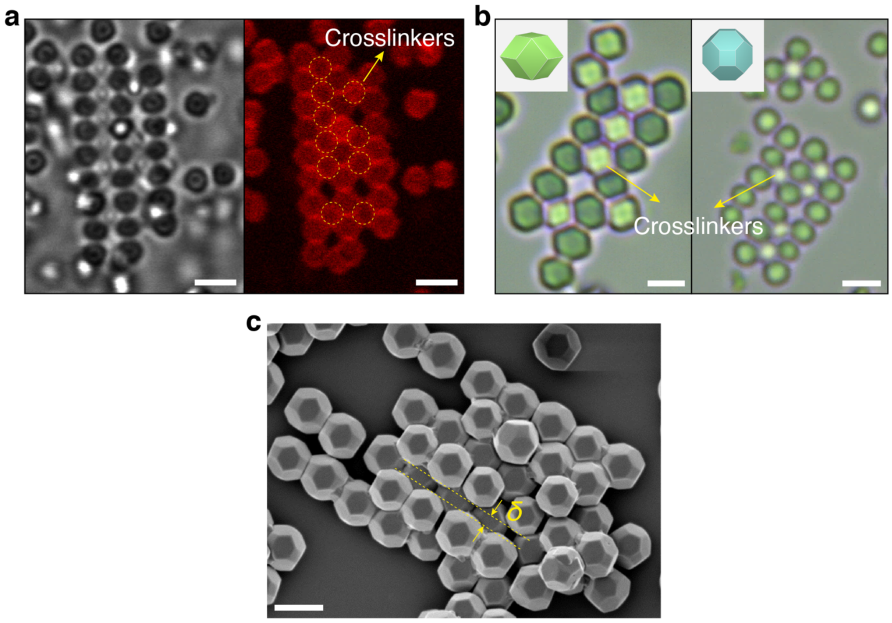

Supplementary Figure 8. Visualization of the crosslinkers of ZIF-8 chain bundles. a, Confocal microscope images of ZIF-8 chain bundles assembled from TRD particles of 1.2 ± 0.2 μm. The substrate confinement is introduced in this case. The particles are labeled with Rhodamine B. The orange circles highlight the crosslinkers between the chains. **b**, Optical microscope images of ZIF-8 chain bundles assembled form RD (left, ~2 μm) and TRD (right, 1.2 ± 0.2 μm) particles. The crosslinkers (indicated by arrows) can be clearly distinguished by the bright dots due to the optical contrast in different layer. **c**, SEM image of 0.8-μm TRD ZIF-8 chain bundles. The chains are separated by a distance of δ ,which equals to the side length of square (100) facet of TRD particles. Scale bars: 2 μm (**a**-**b**) and 1 μm (**c**).

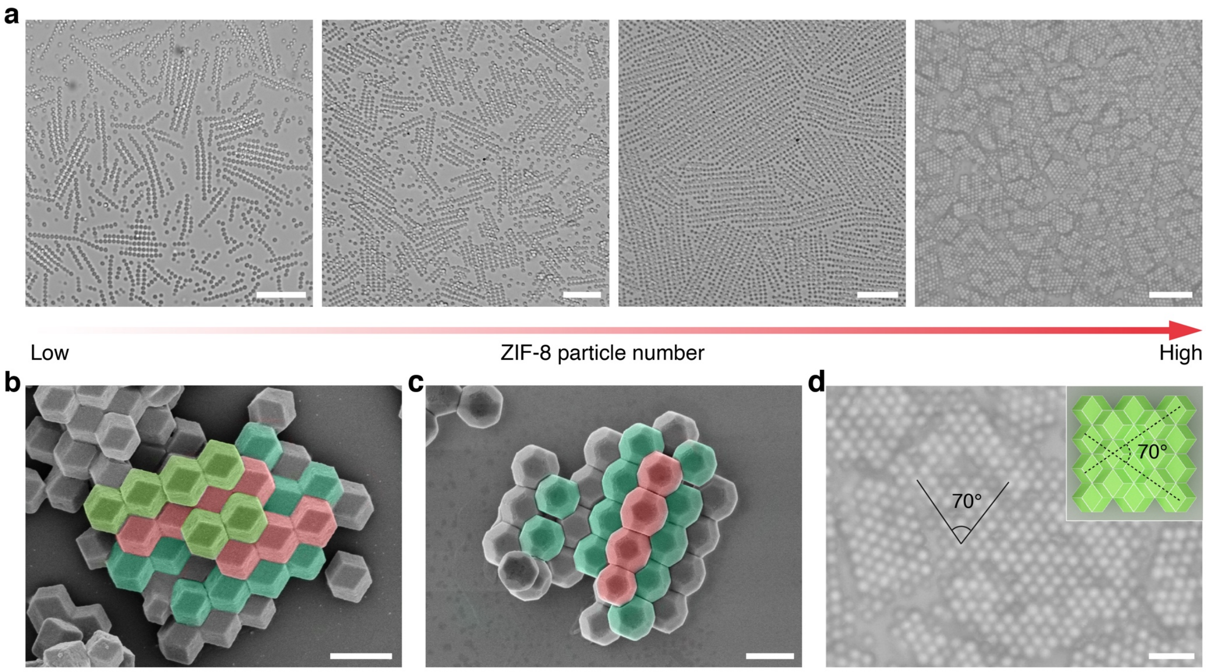

Supplementary Figure 9. Concentration effect on the ZIF-8 colloidal superstructures. a, Optical microscope images show linear chains, small bundles, ordered 2D and quasi-3D crystals (from left to right) by increasing the particle concentration of ZIF-8 (TRD) (specific concentration in Supplementary Table 1). The assemblies are formed on a smooth substrate. **b**-**c**, SEMs of RD (**b**, 0.9 ± 0.1 μm) and TRD (**c**, 0.8 ± 0.1 μm) ZIF-8 quasi-3D crystals. Particles in different layers are false colored. **d**, Magnified microscope image of (**a**) and cartoon (inset) show the quasi-3D crystals with an angle of 70°, representing the (110) particle orientation. Scale bars: 10 μm (**a**), 1 μm (**b**-**c**) and 3 μm (**d**).

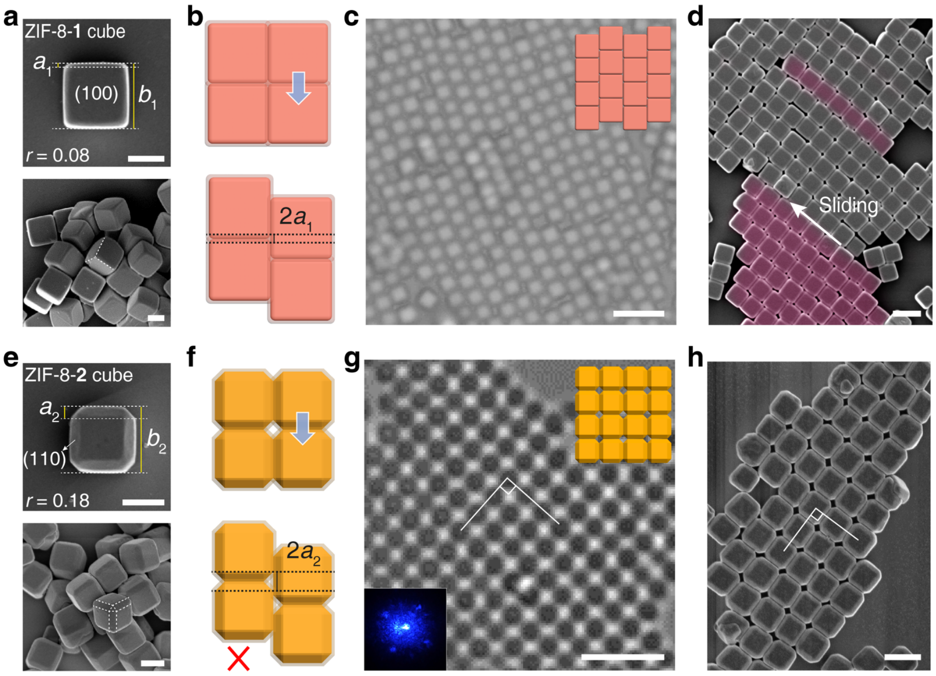

Supplementary Figure 10. 2D films self-assembled from ZIF-8 cubic particles. a, SEM images of cubic ZIF-8-**1** particles with small truncation (defined as $r = a/b$). The representative (100) face is shown. Yellow lines show the dimension of a and b. The smooth edge with slightly rounded corner is highlighted with dashed lines (**a**, bottom image). **b**, Cartoons illustrate the sliding (blue arrow) between two particle rows, which decreases the particle surface overlap approximately by $2a_1(b_1 - 2a_1)$. **c-d**, Bright-field optical image (**c**), cartoon (**c**, inset) and SEM (**d**) showing the superstructures assembled from cubic ZIF-8-**1** particles. Different particle rows (false-colored with mulberry) can slide relative to each other (see Supplementary Note 1). **e**, SEM images of ZIF-8-**2** cubes with large truncation ($r = 0.18$). The sharp edges of beveled (110) facets are highlighted with dashed lines (**e**, bottom). **f**, Cartoons demonstrate that the sliding between particle rows is prohibited by the significant decreases in particle surface overlap, $2a_2(b_2 - 2a_2)$. **g-h**, Square lattice assembled from ZIF-8-**2** cubes. Insets in (**g**) are the corresponding laser diffraction pattern and cartoon showing the superlattice structure. Scale bars: 0.5 µm (**a**, **e**), 3 µm (**c**, **g**) and 1 µm (**d**, **h**).

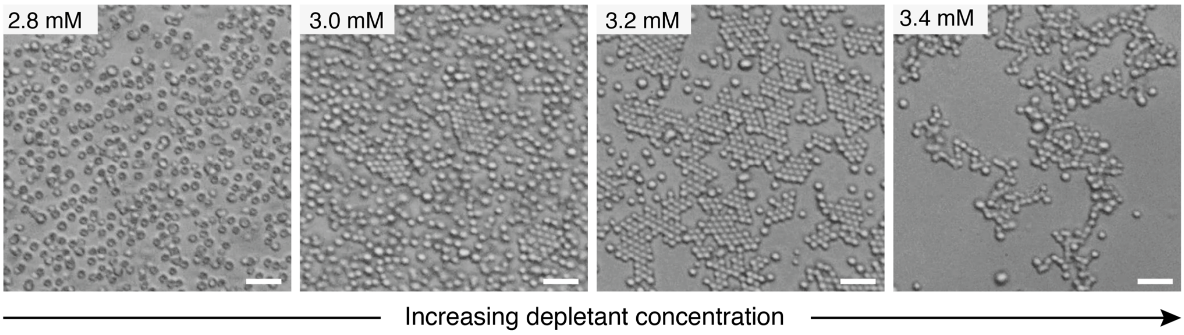

Supplementary Figure 11. The effect of depletant concentration to the self-assembly of UiO-66 particles. Bright field microscope images showing the self-assembly of UiO-66 particles at different depletant (CTAC is used in this case) concentrations. The images were taken at the same assembly time (2 hours). The particle binding is weak to form superlattices at 2.8 mM of CTAC; small nuclei are formed at higher concentration (3.0 mM); 3.2 mM of CTAC accelerates the crystallization process; while 3.4 mM of CTAC is strong to produce porous structures where particles are randomly aggregated. Scale bars: 5 μm.

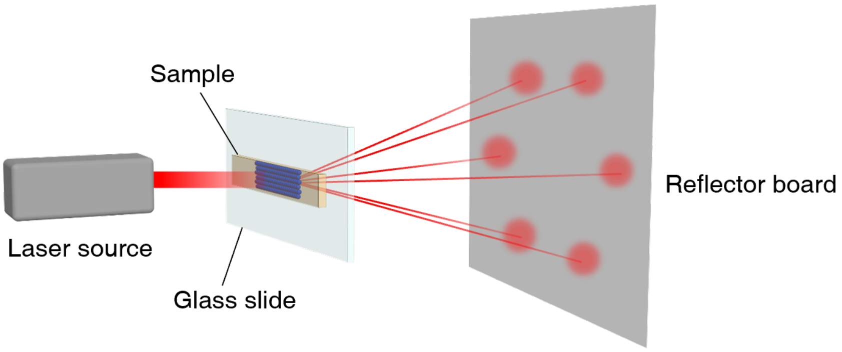

Supplementary Figure 12. Cartoon showing the home-built laser diffraction apparatus. The samples (blue) are sealed in a glass capillary tube (yellow) and mounted on a glass slide (light blue).

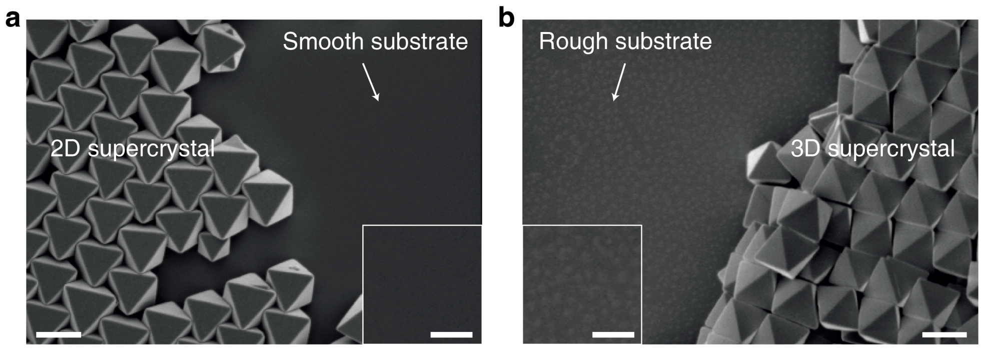

Supplementary Figure 13. Comparison of UiO-66 superstructures on smooth and rough substrates. a-b, SEM images showing the smooth (**a**) and rough (**b**) substrates where the UiO-66 particles form 2D/3D supercrystals, respectively. The insets are the zoom-in SEM images highlighting the surface morphology of the substrates. Scale bars: 1 μm and 0.5 μm (inset).

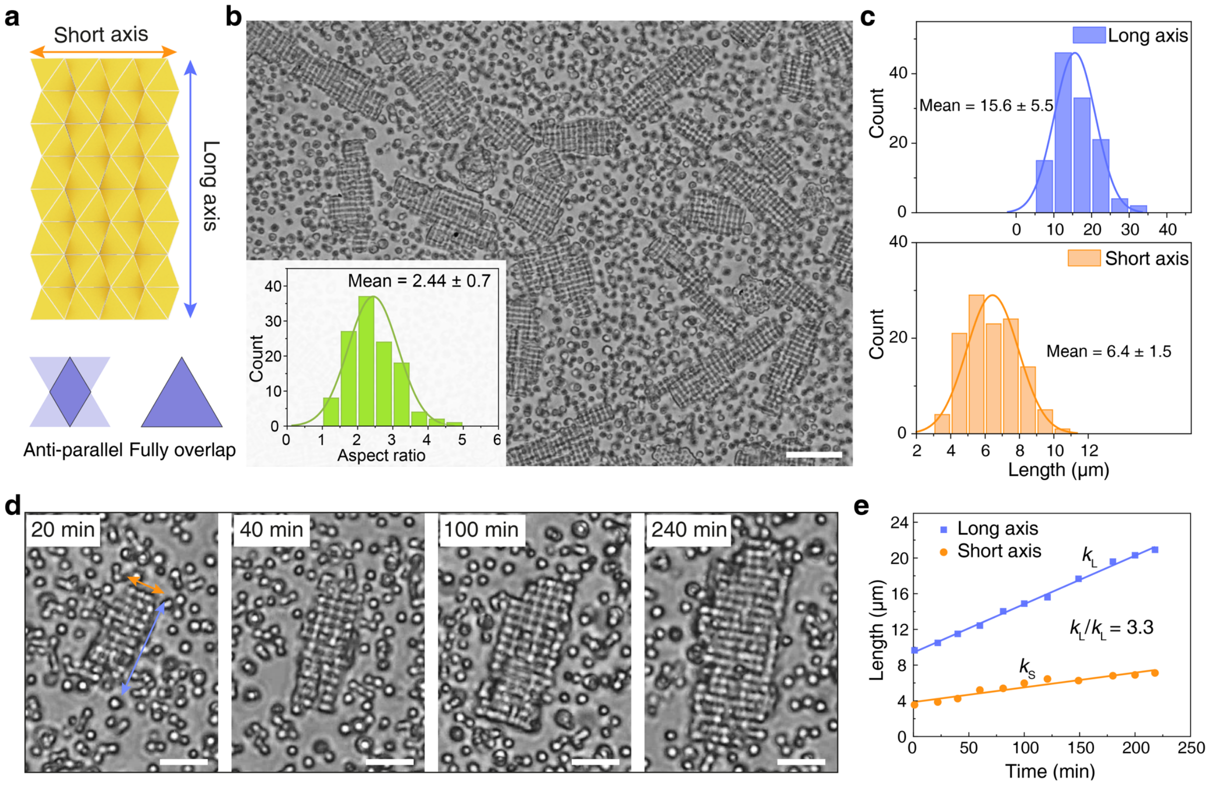

Supplementary Figure 14. Anisotropic/directional growth of UiO-66 quasi-1D stripe-like supercrystals. a, Cartoon of the quasi-1D stripe-like superstructure assembled from UiO-66 particles on the rough substrate. The facet overlap of the particles along the short axis is of an anti-parallel pattern (bottom left) and along the long axis is a fully overlapped pattern (bottom right). **B-c**, Large microscopic view of the assembled structures (**b**), the distribution histograms of the crystals' aspect ratio (**b**, inset) and the lengths of long/short sides (**c**) of different crystals. **d-e**, Snapshots from a video (**d**) show the directional growth of anisotropic UiO-66 superstructures, whose short and long sides in length are plot as a function of time. The growth rate along the long axis (k_L, purple line) is 3.3 times of the one along short axis (k_S, orange line). Scale bars: 10 μm (**b**) and 5 μm (**d**).

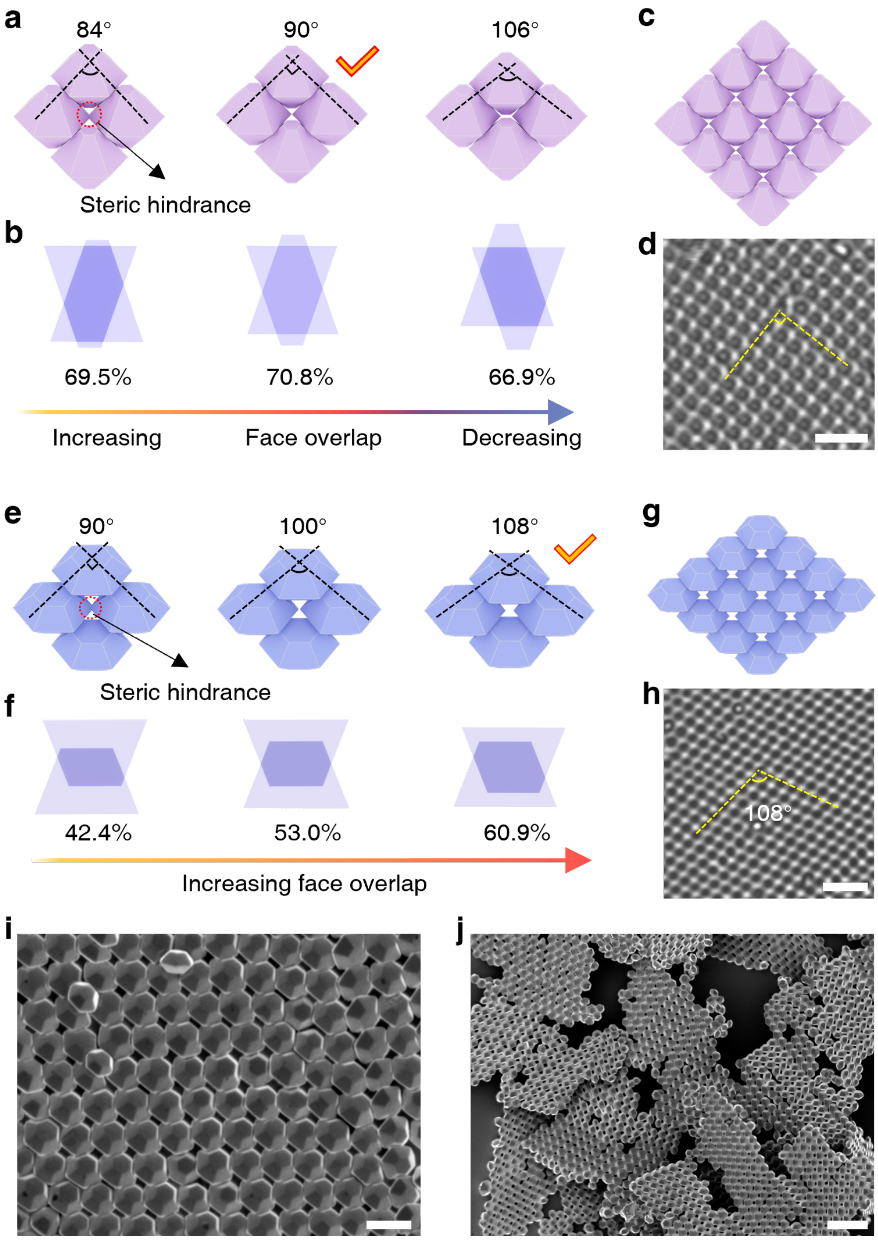

Supplementary Figure 15. The binding and face contact patterns of MIL-96 particles. a-d, Hypothetical unit cells of MIL-96-**1** superlattice with increasing axial angles from 84°, 90° to 106° in (**a**) and their corresponding face overlaps in (**b**). MIL-96-**1** particles prefer to form a square lattice with a maximum face overlap (70.8%), whose structure is represented by the cartoon (**c**) and the optical microscope image (**d**). Lattice of 84° is prohibited by the steric hindrance (**a**, red circle) between particles. **e-j**, The unit cells and facet overlaps of MIL-96-**2** superlattice having axial angles 90°, 100°, and 108° in (**e**, **f**). Similarly, MIL-96-**2** particles tend to maximize the face overlap (60.9%) to form the orthorhombic lattice of 108°, which is shown

by the cartoon in (**g**) and optical microscope image in (**h**). SEMs in (**i**, **j**) show large pieces of MIL-96-**2** orthorhombic lattices attached or peeled from the substrate. Scale bars: 3 μm (**d**, **h**, **j**) and 1 μm (**i**).

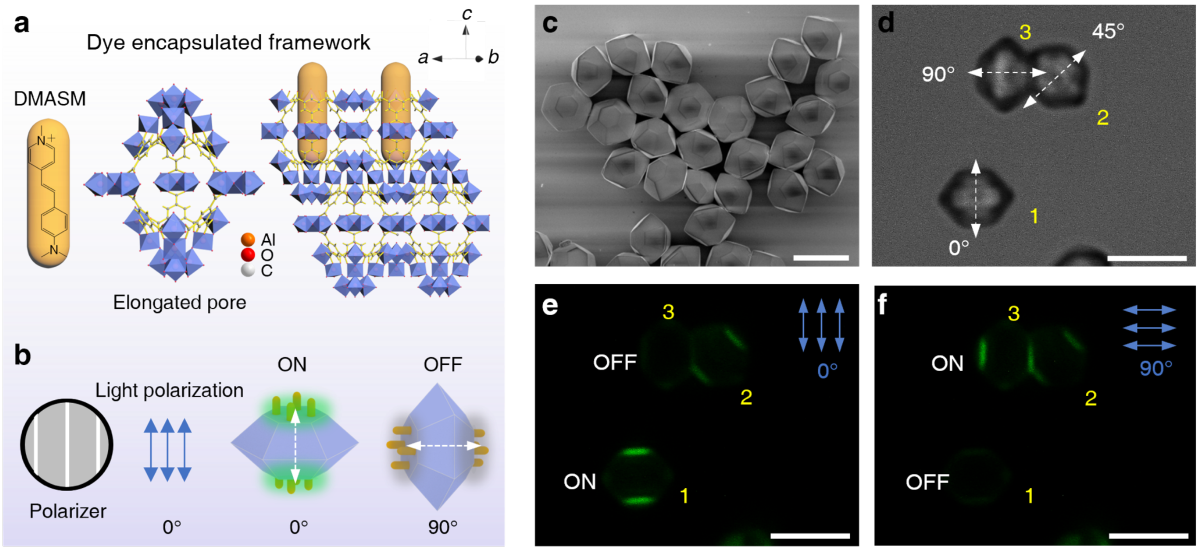

Supplementary Figure 16. Facet-selective encapsulation of DMASM and fluorescence anisotropy on MIL-96 particles. a, Schematics depicting the rod-like structures of DMASM dye molecule (orange rod) and the elongated ellipsoidal pore of MIL-96 along the [002] direction (*c* axis). Atom color code: Al, green; O, red; and C, grey. A possible mechanism of selective dye encapsulation on (002) facets is that the dye molecules enter the pores and align their long axes along the *c* axis. The dye molecules cannot diffuse further inside due to the narrow MOF channels (**a**, right). **b**, When the dye-doped particles are illuminated by linearly polarized light, they show fluorescence anisotropy. The fluorescence turns "on" or "off" when the orientation of dye molecules is parallel (0°) or perpendicular (90°) to the light polarization. This experiment confirms that the dyes are mutually aligned. Blue arrows show the polarization direction of the excitation light. White dotted arrows represent the orientation of dye molecules

on a MOF particle. **c**, SEM image of 4.5-µm MIL-96 particles. **d**, Optical microscope image of three dye-doped MIL-96 particles with different dye orientations (0°, 45°, 90° for particle **1**, **2**, **3**). **e**-**f**, Fluorescence of MIL-96 particles under polarized light (blue arrows, polarization: 0° for **e** and 90° for **f**). Their intensities depend on the particle orientation with respect to the polarization direction (see also Supplementary Video 11). Scale bars: 5 µm.

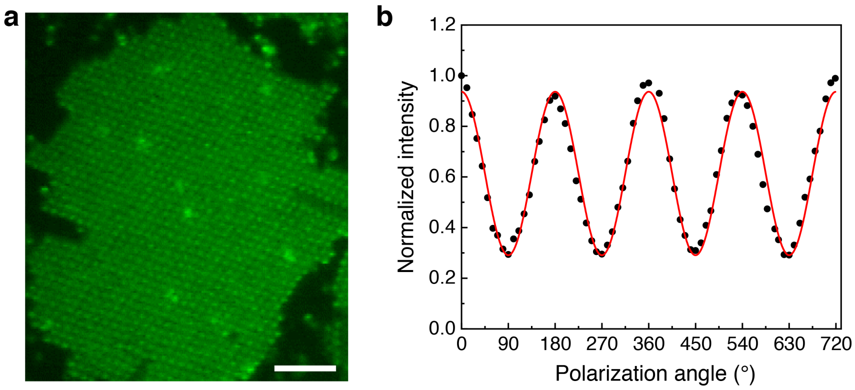

Supplementary Figure 17. Fluorescence anisotropy of dye-encapsulated MIL-96 films. a, Fluorescent microscope image of a MIL-96-**2** film, whose intensity is recorded in cycles of tuning the direction of linearly polarized light. **b**, Plot of normalized intensity versus polarization angles. The fluorescence anisotropy can be repeated many times (see Supplementary Video 12). Scale bar: 5 µm.

Supplementary Tables

Supplementary Table 1. The assembly conditions (particle and depletant concentration) of MOF particles on smooth and rough substrates.

particles	labels	sizes (μm)	depletant	depletant con. (mM)	particle No. (Per screen)*	assembled structures†
ZIF-8	TRD	1.2 ± 0.2	CTAC	5.0 ± 0.2	4500	chains (0.2×), bundles (1×), ordered bundles (2×), quisi-3D crystal (4×)
	RD	2.6 ± 0.3	CTAC	4.0 ± 0.2	1500	chains
		0.9 ± 0.1			5000	same as TRD; quasi-3D/3D crystal (rough substrate)
	ZIF-8-**1** cube	0.5 ± 0.1	CTAC	3.5 ± 0.2	4500	lattice with row sliding
	ZIF-8-**2** cube	0.6 ± 0.1	CTAC	3.9 ± 0.2	6000	square lattice
MIL-88A	-	1.6 ± 0.1	SDS	15.0 ± 3.0	3500	top-down alternating chain; snowflake network (rough substrate)
UiO-66	-	1.0 ± 0.1	CTAC	3.0 ± 0.2	3500	hexagonal lattice; quasi-1D stripe-like structure (rough substrate)
MIL-96	MIL-96-**1**	1.0 ± 0.1	CTAC	3.2 ± 0.2	8000	square lattice

	MIL-96-**2**	0.8 ± 0.1	CTAC	4.4 ± 0.2	7000	centered rectangular lattice

*The particle concentration (1×) used above in a typical self-assembly is the estimated particle number per 2D area (157 μm × 89 μm).

†All superstructures are assembled on a smooth substrate, unless otherwise specified.

Supplementary Table 2. Zeta potentials of different MOF particles in water with/without ionic amphiphiles.

**particles**	**Zeta potential (mV)**		
	**DI water**	**with ionic amphiphiles***	
ZIF-8	+15.8 ± 0.54	CTAC, 5.0 mM	+56.0 ± 0.78
MIL-88A	+16.9 ± 0.19	SDS, 15 mM	-60.9 ± 0.76
UiO-66	+23.3 ± 2.69	CTAC, 5.0 mM	+37.9 ± 2.45
MIL-96	-16.5 ± 0.21	CTAC, 5.0 mM	+35.5 ± 0.65

*All particles are mixed with the ionic amphiphiles solutions for 2 hours before the measurement.

Supplementary Notes

Supplementary Note 1. Self-assembly of ZIF-8 particles with cubic shapes.

We also show that ZIF-8 particles with cubic shapes self-assemble into 2D square lattices, whereby each particle is oriented to the substrate by its (100) square facet while packing with four neighboring particles (Supplementary Fig. 10). Subtle difference in shape due to truncation, defined as $r = a/b$, leads to different structures. For standard cubes ZIF-8-**1** with slightly rounded edges ($r = 0.08$), a row of particles within the 2D lattice can slide relative to each other (Supplementary Fig. 10a-d, Supplementary Video 4). In this case, because the vertices of the

cubes are well-fitted with little interstitial space, the sliding does not appreciably alter the overlap volume between the bound particles. However, for the much-truncated ZIF-8-**2** cubes (r = 0.18), which feature beveled (110) facets, smaller (100) facets, and sharp edges, they precisely align with four neighbors creating a square lattice, as shown by optical and electron microscopy and revealed by laser diffraction pattern (Supplementary Fig. 10e-h). The row sliding is energetically prohibited, as it would significantly decrease the overlap between the (100) facets of the particles by a factor of 56 %, as we discuss below (Supplementary Fig. 10f and Supplementary Video 3-4).

The depletion attraction energy between two cubic particles due to a number density n of depletant can be estimated by:

$$\Delta G_{\mathrm{dep}} = -nk_{\mathrm{B}}T\Delta V, \qquad \text{(Supp. Eq. 1)}$$

where the overlap of exclusion volume ΔV for cubes is approximated by[1]:

$$\Delta V \approx (b - 2a + d)^2 d, \qquad \text{(Supp. Eq. 2)}$$

where d is the diameter of depletants (CTAC micelle), b is the cube's side length and a is the truncated length of the cube.

The relative sliding rows of particles will decrease the overlap volume by:

$$\delta \approx (2a - d)(b - 2a + d)d, \qquad \text{(Supp. Eq. 3)}$$

Thus, the degree of decreased overlap volume can be estimated by:

$$\frac{\delta}{\Delta V} = \frac{(2a - d)(b - 2a + d)d}{(b - 2a + d)^2 d} \qquad \text{(Supp. Eq. 4)}$$

which can be simplified and approximated by:

$$\frac{\delta}{\Delta V} = \frac{2a - d}{b - 2a + d} \approx \frac{2(a/b)}{1 - 2(a/b)}, \qquad \text{(Supp. Eq. 5)}$$

where (*a/b*) denotes the extent of truncation *r*.

For cubic ZIF-8 particles with different truncations ($r = a/b = 0.08$ and 0.18), the overlap volume between two particles is reduced by 19% and 56%, respectively. As a result, the particle row sliding is energetically prohibited for the latter one ($r = 0.18$).

Supplementary References

1 Rossi, L. *et al.* Cubic crystals from cubic colloids. *Soft Matter* **7**, 4139-4142, (2011).
